# Myoclonus and other jerky movement disorders

**DOI:** 10.1016/j.cnp.2022.09.003

**Published:** 2022-10-06

**Authors:** Sterre van der Veen, John N. Caviness, Yasmine E.M. Dreissen, Christos Ganos, Abubaker Ibrahim, Johannes H.T.M. Koelman, Ambra Stefani, Marina A.J. Tijssen

**Affiliations:** a Department of Neurology, University of Groningen, University Medical Centre Groningen (UMCG), Groningen, The Netherlands; b Expertise Centre Movement Disorders Groningen, University Medical Centre Groningen (UMCG), Groningen, The Netherlands; c Department of Neurology, Mayo Clinic Arizona, Movement Neurophysiology Laboratory, Scottsdale, AZ, USA; d Department of Neurosurgery, Amsterdam University Medical Centers, Amsterdam, The Netherlands; e Movement Disorders and Neuromodulation Unit, Department of Neurology, Charité University Medicine Berlin, Berlin, Germany; f Department of Neurology, Medical University of Innsbruck, Innsbruck, Austria; g Department of Neurology and Clinical Neurophysiology, Amsterdam University Medical Centers, Amsterdam, The Netherlands

**Keywords:** Neurophysiology, Myoclonus, Tics, Tourette disorder, Startle, RLS, PLMS, EEG, EMG, Transcranial Magnetic Stimulation, Local field potentials, Deep Brain Stimulation

## Abstract

Myoclonus and other jerky movements form a large heterogeneous group of disorders. Clinical neurophysiology studies can have an important contribution to support diagnosis but also to gain insight in the pathophysiology of different kind of jerks. This review focuses on myoclonus, tics, startle disorders, restless legs syndrome, and periodic leg movements during sleep. Myoclonus is defined as brief, shock-like movements, and subtypes can be classified based the anatomical origin. Both the clinical phenotype and the neurophysiological tests support this classification: cortical, cortical-subcortical, subcortical/non-segmental, segmental, peripheral, and functional jerks. The most important techniques used are polymyography and the combination of electromyography-electroencephalography focused on jerk-locked back-averaging, cortico-muscular coherence, and the Bereitschaftspotential. Clinically, the differential diagnosis of myoclonus includes tics, and this diagnosis is mainly based on the history with premonitory urges and the ability to suppress the tic. Electrophysiological tests are mainly applied in a research setting and include the Bereitschaftspotential, local field potentials, transcranial magnetic stimulation, and pre-pulse inhibition. Jerks due to a startling stimulus form the group of startle syndromes. This group includes disorders with an exaggerated startle reflex, such as hyperekplexia and stiff person syndrome, but also neuropsychiatric and stimulus-induced disorders. For these disorders polymyography combined with a startling stimulus can be useful to determine the pattern of muscle activation and thus the diagnosis. Assessment of symptoms in restless legs syndrome and periodic leg movements during sleep can be performed with different validated scoring criteria with the help of electromyography.

## Highlights

• This review focuses on myoclonus, tics, startle syndromes, restless legs syndrome and periodic leg movements during sleep.
• Tools that can be used to study movement include kinematics, kinetics, and the underlying muscle activity with EMG.
• The brain activity driving movement can be studied with EEG, MEG, and functional MRI.

This chapter summarizes the clinical neurophysiology of myoclonus and other jerky movements, such as tics, startle disorders, the restless legs syndrome, and periodic leg movements during sleep. It consists of a heterogenous group of disorders but they may be difficult to distinguish. For both the naïve and experienced witness, the very brief duration of occurrence of the motor event does not allow the observer to subjectively analyze the entire jerk. As soon as it occurs, it has already disappeared. Useful tools to support the clinicians include video recordings and objective electrophysiological tests, of which the latter are discussed below.

## 1 Myoclonus

### 1.1 Introduction

#### 1.1.1 Definition

**Fig. 1:**
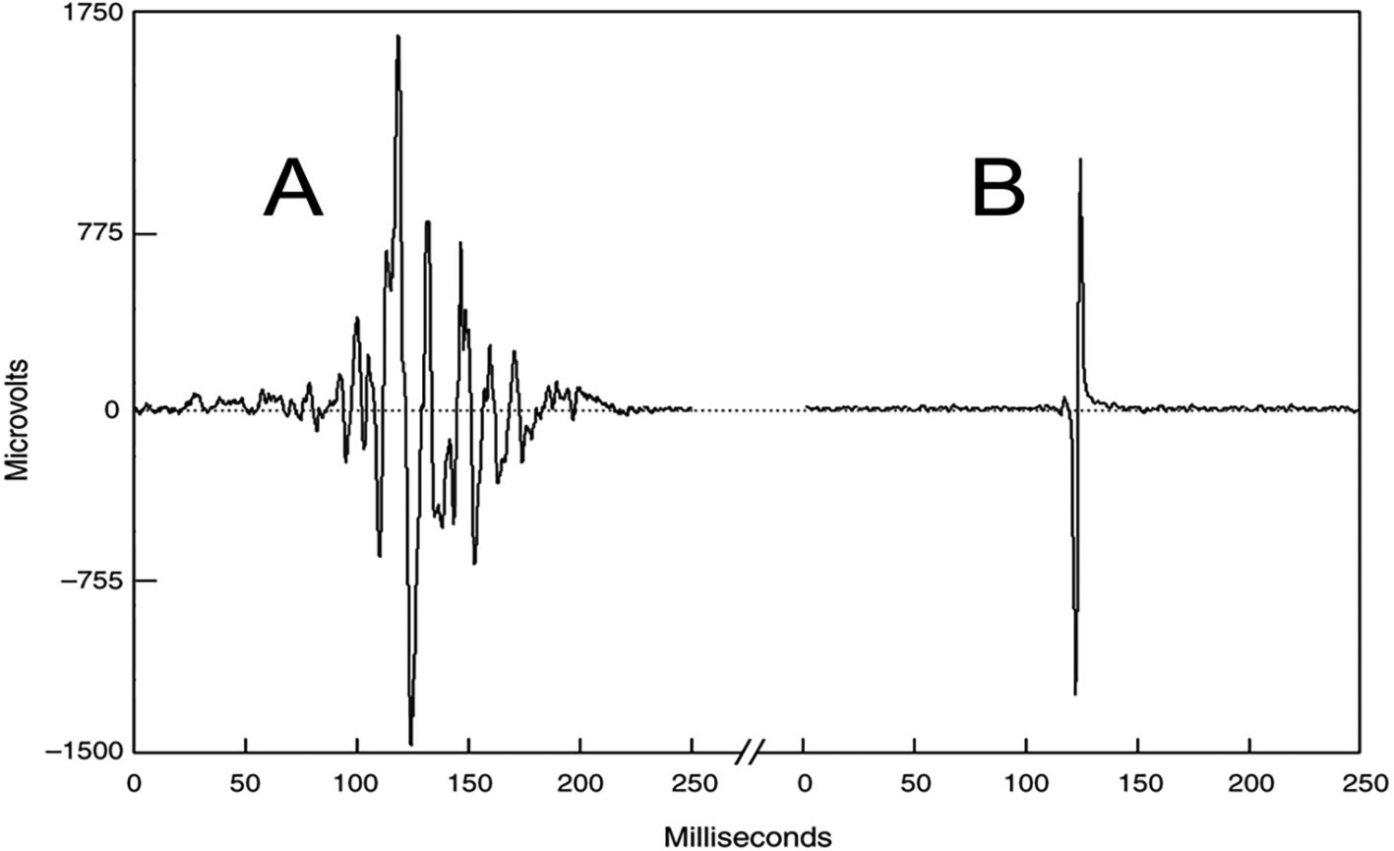
A shows the surface electromyographic (EMG) pattern from a normal voluntary ballistic movement of wrist flexion. The subject was instructed to perform the wrist extension as quick and as brief as possible. In B, there is a myoclonus surface EMG wrist extension discharge from a patient with multifocal action myoclonus. Despite the fact that the normal ballistic movement was performed as brief as possible, note that there is still a more gradual build-up of activity when compared to the involuntary myoclonus EMG discharge. Modified from (Caviness, 1996).

Myoclonus, as a hyperkinetic movement disorder phenotype, represents the briefest jerk produced by abnormal neuromuscular activation of central nervous system origin. Myoclonus is clinically defined as sudden, brief, shock-like, involuntary movements caused by muscular contractions or inhibitions (Caviness, 2019). On electromyography (EMG), myoclonus appears as a brief discharge that is usually so synchronous as to have a very different appearance from a voluntary ballistic EMG discharge (Fig. 1). It has a distinctive monophasic feature, with only one component of the movement being active followed by a passive return to baseline. This sudden firing of neurons that causes or affects neuromuscular activation can originate from a variety of nervous system levels and locations. As a result, it should be expected that different physiologies can produce myoclonus with associated electrophysiological signatures on EEG and EMG. It also follows that multiple anatomical locations can originate myoclonus if pathologically altered. Moreover, a wide variety of diseases and conditions may create a type of neuronal dysfunction that causes myoclonus (Caviness and Brown, 2004).

#### 1.1.2 Syndrome classification and etiologies

A clinical classification scheme assists with diagnosis by providing high-level syndrome characteristics and associate them with specific etiologies. The Marsden-Hallett-Fahn classification scheme is commonly used. The major categories in this scheme are: physiologic, essential, epileptic and symptomatic (secondary) (Marsden et al., 1982). Each of the major categories is associated with different clinical presentations.

A) Physiologic myoclonus is a normal phenomenon. There is minimal or no associated disability from this, and the physical exam reveals no relevant abnormality. Jerks during sleep are the most familiar examples of physiologic myoclonus.
B) Essential myoclonus refers to myoclonus that is a primary or only clinical finding. Essential myoclonus is idiopathic sporadic or hereditary and progresses slowly or not at all. Many families with hereditary essential myoclonus manifest a genetic mutation in the epsilon-sarcoglycan gene. Dystonia can be a prominent component in these patients.
C) Epileptic myoclonus refers to the presence of myoclonus in the setting of a chronic seizure disorder (epilepsy). Myoclonus can occur as only one component of a seizure, the only seizure manifestation, or one of multiple seizure types within an epileptic syndrome (Caviness, 2011). The most common example of this category is the juvenile myoclonic epilepsy of Janz which is a type of idiopathic generalized epilepsy.
D) Symptomatic (secondary) myoclonus manifests in the setting of an identifiable underlying disorder, neurologic or non-neurologic. Common associations in this category are cognitive changes and ataxia. Symptomatic causes of myoclonus comprise a widely diverse group of disease processes and include neurodegenerative diseases, storage diseases, toxic-metabolic states, diffuse brain physical injuries, infections, inflammation, focal nervous system damage, and paraneoplastic syndromes as well as other medical disorders (Pena and Caviness, 2020).

Most clinically relevant cases of myoclonus are in the symptomatic category, followed by the epileptic and essential categories (Caviness et al., 1999). In an epidemiological study, myoclonus was found to have an average annual incidence of 1.3 cases per 100,000 with a prevalence of 8.6 per 100,000 person-years (Caviness et al., 1999). The specific etiologies under each category have been updated over time (Pena and Caviness, 2020).

#### 1.1.3 Evaluation

Evaluation should be determined by features of the history, physical exam, and clinical category classification (Caviness, 2019, Caviness and Brown, 2004, Zutt et al., 2015). A systematic approach leading the clinician through the diagnostic steps was made by Zutt et al (2015). Special attention should be given to the presence of concomitant medical conditions, family history of similar problems, and exposure to toxins and drugs known to cause myoclonus. If a drug is suspected to be causative for a patient’s myoclonus, consideration should be given to cautiously decreasing or discontinuing the medication. Thus, the result of the medication change may be therapeutic as well as diagnostic. When the cause of the myoclonus is unexplained after this initial evaluation, the following minimal basic testing should be performed: laboratory testing (electrolytes & glucose, renal and hepatic function tests, calcium, magnesium), paraneoplastic testing, drug and toxin screen, electroencephalography, and brain imaging.

This testing mainly evaluates acquired causes of myoclonus such as metabolic, toxic, structural brain lesions, seizure disorders, and cancer related causes. If these tests do not reveal the diagnosis, then more advanced testing should be considered. This testing may include cerebrospinal fluid examination, enzyme activity, imaging for cancer, tissue biopsy, and other tests (Pena and Caviness, 2020). These days genome testing is much more available and therefore, could be considered at an earlier stage, once acquired forms have been excluded (Veen et al., 2019, Zutt et al., 2015). Before genetic testing is done, the patient should be fully aware of the implications for both positive and negative results. If appropriate, genetic counseling is recommended.

There is no point in the evaluation of myoclonus where electrophysiological testing does not add value. Especially, if the etiology of the myoclonus is not obvious, electrophysiological testing should be seriously considered. This value comes from the electrophysiological evaluation being able to define both anatomy and physiology. The definition of the anatomy assists with assigning etiology, and the definition of the physiology assists with both assigning etiology and treatment approach.

### 1.2 Neurophysiology classification of myoclonus

#### 1.2.1 General concepts and methods

The sudden, brief jerk of myoclonus has led naturally to the idea that it is generated from a source which drives hyperexcitable drive to motor neurons which activates a muscle jerk in the case of positive myoclonus. The neurophysiological classification of myoclonus predominantly takes a physiological approach, but importantly, the anatomical source within the nervous system is reflected in the neurophysiology classification category. Electrophysiological testing applied in the neurophysiological classification of myoclonus usually includes multichannel surface electromyography (EMG) recording, long latency EMG responses to mixed nerve stimulation, electroencephalography (EEG), EEG-EMG polygraphy with back-averaging, and evoked potentials (e.g., median nerve stimulation somatosensory evoked potential (SEP) (Caviness, 2003, Chen and Chen, 2020, Shibasaki, 2000, Zutt et al., 2018). Cortico-muscular coherence is used as additional analysis method (Brown et al., 1999). Positive and negative findings from these methods are used to determine the physiological type of myoclonus (Caviness, 2003, Zutt et al., 2018). The different physiological types of myoclonus are organized into a classification scheme reflecting physiological generation. The main physiological categories for myoclonus classification are (Caviness, 2003, Pena and Caviness, 2020):

- Cortical
- Cortical-Subcortical
- Subcortical/non-segmental
- Segmental
- Peripheral
- Functional

Further subdivision can be made on the basis of additional electrophysiological testing. Multiple myoclonus physiology types can occur in the same patient. The individual categories of the physiological classification scheme will be discussed.

#### 1.2.2 Cortical Myoclonus

##### 1.2.2.1 Cortical myoclonus in clinical practice

Cortical physiology is the most common physiology for myoclonus. The precise alteration in neuronal physiology that produces the cortical motor circuit hyperexcitability is unknown. Defects in lack of inhibition and/or abnormal modulation of excitation have been proposed. However, etiologies that manifest cortical myoclonus often are associated with pathology not only in the cortex, but also diffuse brain pathology. The influence of other brain sites, such as the cerebellum has been speculated, but evidence remains based on correlation only and yet to be confirmed (Latorre et al., 2020).

Clinically, cortical myoclonus patients have one or more of three clinical manifestation syndromes. First, cortical myoclonus is associated with reflex activation, in association usually with muscle activation or “action myoclonus.” However, because of the reflex features, this is referred to as “cortical reflex myoclonus” (Shibasaki, 2000). Second, cortical myoclonus may be present without reflex activation. This is common in small amplitude muscle activation myoclonus. Third, the clinical manifestation may be focal motor seizures including epilepsia partialis continua. In any of these syndromic manifestations, the distribution of the myoclonus may be focal, segmental, multifocal, bilateral, and even generalized. Any combination of activation patterns from rest, action, or reflex may be seen. Since a variety of distributions and activation characteristics are possible with cortical myoclonus, it highlights the importance of defining the neurophysiology via the electrophysiological characteristics.

For the purposes of both diagnosis and treatment, clinicians are recommended to determine if the myoclonus is cortical origin (Pena and Caviness, 2020, Zutt et al., 2018). There are many heterogeneous etiologies of cortical myoclonus. Cortical myoclonus may be acquired or genetic. The best known causes for cortical myoclonus include post-hypoxic syndrome, progressive myoclonus ataxia/epilepsy syndromes, toxic-metabolic (including medications), and neurodegenerative syndromes which are often associated with cognitive problems. This list is not complete, and it should be anticipated that new diagnoses will have cortical myoclonus described. In the paper by Zutt et al, the clinician is guided with a systematic approach how to reach an etiological diagnosis (Zutt et al., 2015). Moreover, the clinician should be aware that multiple neurophysiology types of myoclonus are possible in the same disorder and even the same patient.

The presence of cortical myoclonus suggests a known treatment strategy that leverages anti-seizure medications (Dijk and Tijssen, 2010, Pena and Caviness, 2020). This makes sense due to the likely parallels between myoclonus and seizure pathophysiology. Levetiracetam is generally regarded as first-line treatment for cortical myoclonus. Valproic acid and clonazepam are also used in cortical myoclonus. Unfortunately, it is common for therapeutic responsiveness to be limited and complicated by side effects. Sodium oxybate has seen increasing evidence for its use, although is usually reserved after other medications have been tried (Riboldi and Frucht, 2019). Polypharmacy is often necessary. Recently, perampanel has been suggested in cortical myoclonus in progressive myoclonic epilepsy syndrome, as well as for myoclonus in these syndrome for which the pathophysiology is unknown (Kawano et al., 2020). Deep brain stimulation surgery has been gaining increasing study, but it is not known whether it is a reliable therapy. Several stimulation sites have been attempted including thalamus, globus pallidus, subthalamic nucleus, and substantia nigra pars reticulata (di Giacopo et al., 2019, Kobayashi et al., 2010, Ramdhani et al., 2017).

##### 1.2.2.2 General neurophysiology principles for cortical myoclonus

The human homunculus representation within the sensorimotor cortex has the largest contiguous somatotopic representation in the brain. This fact provides an opportunity for widespread hyperexcitability within these sensorimotor circuits to produce sudden, very brief EMG discharges that result in multifocal myoclonus through corticospinal pathways. Depending on the severity of the hyperexcitability in the sensorimotor circuits, segmental and bilateral cortical myoclonus may also occur. The tendency for this hyperexcitability to spread may be associated with multiple muscle area involvement in the same jerk, repetitive myoclonus, and even seizures (Brown et al., 1991a). Sensorimotor cortical areas have agonist and antagonists for a given movement, as well as contiguous muscle areas in close proximity. This produces synchronous or nearly synchronous co-contraction between the muscles involved in a single instant of the jerk.

Cortical origin produces important clinical characteristics for the myoclonus (Caviness, 2003, Caviness and Brown, 2004):

- Since the limbs have the largest representation within the homunculi, the limbs are disproportionally affected in cortical myoclonus.
- Motor areas of the cerebral cortex are critical for voluntary and intentional muscle activation, resulting in action myoclonus predominantly.
- The close proximity of cortical motor and sensory areas provides an opportunity for cross-activation producing sensory reflex myoclonus. The presence of myoclonus induced by reflex stimulation with timing consistent for a transcortical loop is a property consistent with cortical origin myoclonus.

##### 1.2.2.3 Electrophysiological findings seen in cortical myoclonus

The following electrophysiological findings are seen in cortical myoclonus:

a) Brief myoclonus EMG discharges
b) Focal EEG Transient preceding the Myoclonus
c) Enhanced long latency EMG responses to mixed nerve stimulation at rest
d) Enlarged Cortical Somatosensory Evoked Potentials (SEPs)
e) Elevated Cortico-muscular (EEG-EMG) Coherence
f) EEG Seizure discharges
a) Brief myoclonus EMG discharges

**Fig. 2:**
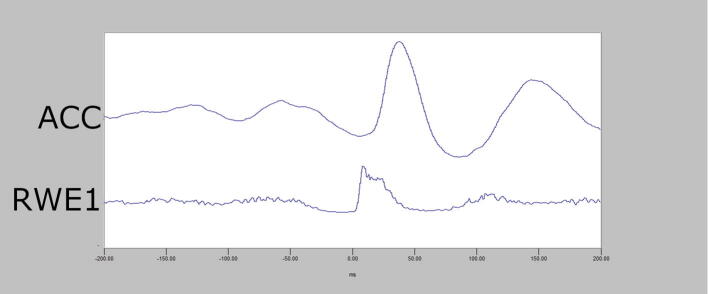
Top: Averaged accelerometer (ACC) deflection. Bottom: Averaged rectified right wrist extensor electromyographic (RWE1) discharge of less than 50 ms. A time-locked relationship between myoclonus electromyographic discharge and the sudden, brief movement, through signal averaging is shown.

**Fig. 3:**
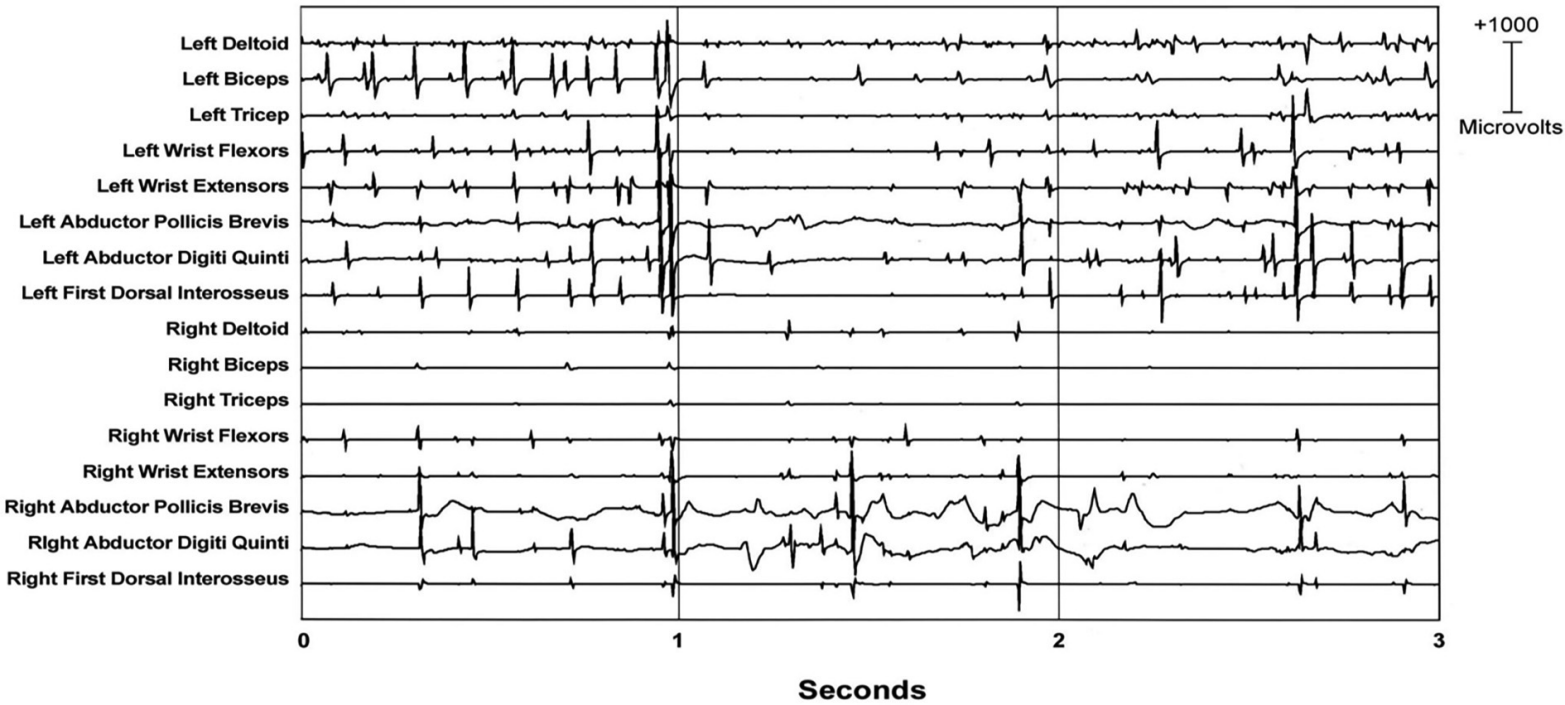
Multichannel surface electromyographic (EMG) recording in upper extremities during postural activation from a patient with cortical myoclonus. There are myoclonus EMG discharges that occur with almost synchronous timing bilaterally.

In cortical myoclonus, EMG discharges appear hypersynchronous, since the multiple motor unit discharge is significantly shorter than typical voluntary ballistic movement discharges which are usually 50–100 ms duration (Caviness and Brown, 2004). EMG discharges are most commonly 25–50 ms duration, although 50–100 ms can be seen (Fig. 2). In Fig. 2, a time-locked correlation is shown between an averaged accelerometer deflection (top trace) and the rectified average of the right wrist extensor EMG of less than 50 ms. For a single cortical myoclonic jerk, surface EMG discharges are almost perfectly synchronous between agonist and antagonist (co-contracting pattern), and also with the nearest contiguous muscle segments. The myoclonus EMG discharges often occur in high-frequency rhythmic bursts or trains. It should be realized that the visual appearance of the myoclonus is usually irregular because there is marked variability between the amplitude of the myoclonus EMG discharges within and between discharge trains. Bilateral EMG discharges with myoclonus may occur, and this is thought to be due to transcallosal spread of the motor excitation (Fig. 3). In Fig. 3, both unilateral and bilateral upper extremity discharges are demonstrated in a cortical myoclonus patient.

If the discharge trains are small and almost continuous, the term “cortical tremor” is often used. In this instance, clinically there is rhythmic oscillation of the distal upper extremities and possibly other locations (Latorre et al., 2020). Cortical tremor is known to be rhythmic cortical myoclonus. It is famously associated with the syndrome, “familial cortical myoclonic tremor and epilepsy (FCMTE)”.

Most myoclonus is “positive,” meaning the jerk results from increased EMG activity that creates a movement in a positive direction stemming from the muscles involved. Negative myoclonus refers to a decrease in tonic EMG activity that creates a brief postural lapse. The term, “asterixis”, refers to negative myoclonus. The EMG silence that creates negative myoclonus has a duration of 50–200 ms. Three types of EMG patterns have been described (Toro et al., 1995, Ugawa et al., 1989). Type I asterixis shows an abrupt offset of EMG silence during voluntary tonic muscle activation. A type II asterixis is associated with a brief, discrete burst of EMG activity that precedes the EMG silence and postural lapse. Type III asterixis follows typical significant positive myoclonus, especially in trains. In this scenario, alternating and consecutive positive and negative jerks occur. The clinical appearance will depend on whether the negative jerks are longer/more prominent that the positive jerks. Most negative myoclonus is cortical, but a subcortical origin cannot ruled out, especially for type I. For repetitive trains of myoclonus, including “cortical tremor”, this type of asterixis is underappreciated. The EMG silences of negative myoclonus are commonly toxic-metabolic, but they can be seen in almost any cause of positive myoclonus, such as in post-hypoxic myoclonus or progressive myoclonic epilepsy. Co-occurrence of positive and negative myoclonus is common.

b) Focal cortical EEG transient preceding myoclonus

**Fig. 4:**
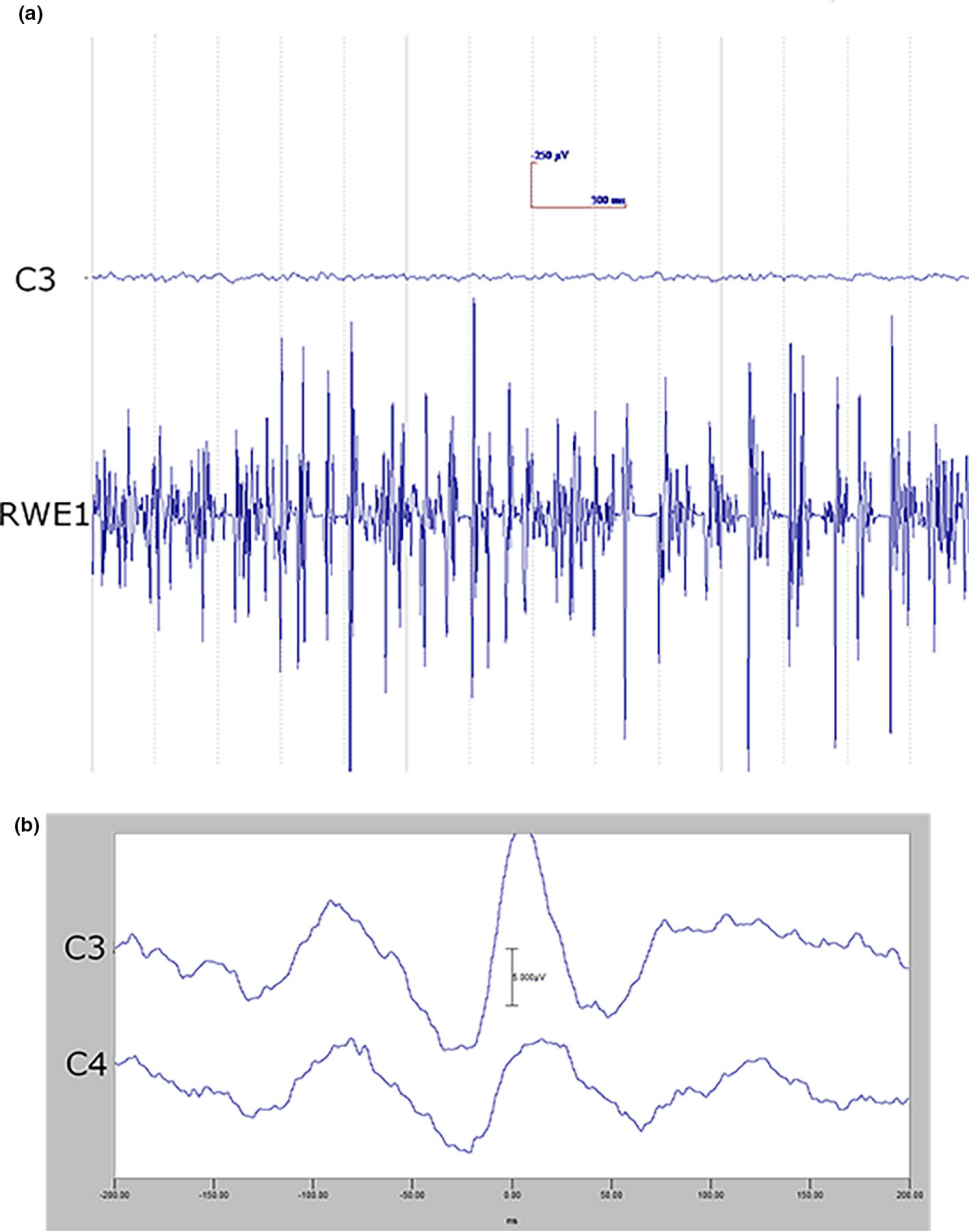
Cortical myoclonus. A: Multiple brief myoclonus electromyographic (EMG) discharges seen in the lower trace during postural activation of the right wrist extensor musculature (RWE1). The contralateral EEG motor area (C3) shows no gross encephalographic (EEG) transient correlate in the upper trace. B: Cortical myoclonus. Using 100 surface myoclonus EMG discharges to perform EEG-EMG back-averaging to increase signal to noise ratio, a sharp EEG transient is elicited at C3. The averaged EEG transient at C4 is smaller, has different configuration, and not significantly different from the C4 EEG waves before and after the trigger (time 0). These findings together demonstrate a cortical myoclonus physiology.

The EEG transient precedes the myoclonus by < 40 ms (arm). Essentially, this constitutes an EEG spike or sharp wave discharge preceding the myoclonus EMG discharge, and if consistently time-locked to the myoclonus, is strongly confirmatory for the existence of cortical myoclonus physiology (Pena and Caviness, 2020, Shibasaki, 2000, Zutt et al., 2018). At times, the pre-myoclonus EEG transient can be observed grossly on the EEG recording. However, EEG back-averaging is a more sensitive and definitive assessment for cortical myoclonus for two reasons: a) the EEG transient is usually lost in the background of the gross EEG. EEG back-averaging increases the signal to noise ratio dramatically, thereby enables the detection of a direct cortical EEG transient that otherwise would not be detected, b) a back-averaged EEG transient also documents a consistent time-locked relationship between cortical discharge and the myoclonus, thereby providing strong evidence for the cortical genesis of the myoclonus (Fig. 4A and B). The transient is localized over the contralateral sensorimotor cortex. The EEG transient is a bi-phasic or tri-phasic spike beginning with a positive deflection whose peak precedes the onset of the myoclonic discharge by an average of 20 ms for arm (range 10–40 ms). The latency between the EEG transient and myoclonus is marked from the positive wave peak of the EEG transient. The myoclonus triggered for EEG back-averaging is detected in two ways: 1) jerk-lock back-averaging refers to having a motion detector as the trigger, or 2) EEG-EMG back-averaging refers to using the onset of the myoclonus EMG discharge as the trigger. Both methods have advantages and disadvantages. Jerk-lock method shows visual correlation with the myoclonus movement itself. The EEG-EMG back-averaging method ensures a specific correlation with the onset of the myoclonus if each EMG discharge trigger produces a myoclonus jerk movement. It is recommended that at least 100 artifact-free epochs are used in the averaging of the myoclonus triggers in order to ensure maximal sensitivity. If the purpose is to demonstrate the absence of a back-averaged transient, then 200 artifact-free triggers are recommended.

In Fig. 4A, the bottom tracing shows right wrist extensor surface EMG with multiple myoclonus EMG discharges. In the top tracing, EEG over the contralateral motor area (C3) shows oscillating EEG signal, but there is no gross presence of EEG transient waves which are correlated with the myoclonus EMG discharges. In Fig. 4B, EEG-EMG back-averaging from 100 myoclonus EMG discharges yielded a time-locked EEG transient maximal at C3.

For negative myoclonus the EEG correlate of type II negative myoclonus may be similar to that of positive cortical myoclonus. The entity, cortical reflex negative myoclonus” was described by Shibasaki and is associated with a silent period after median nerve stimulation and enlarged cortical SEP (Shibasaki et al., 1994). There is evidence that a biphasic transient in the primary motor cortex is involved (Butz et al., 2014). Type I negative myoclonus does not have an EEG correlate and may have a (sub)cortical generator.

c) Enhanced long latency EMG responses to mixed nerve stimulation at rest

**Fig. 5:**
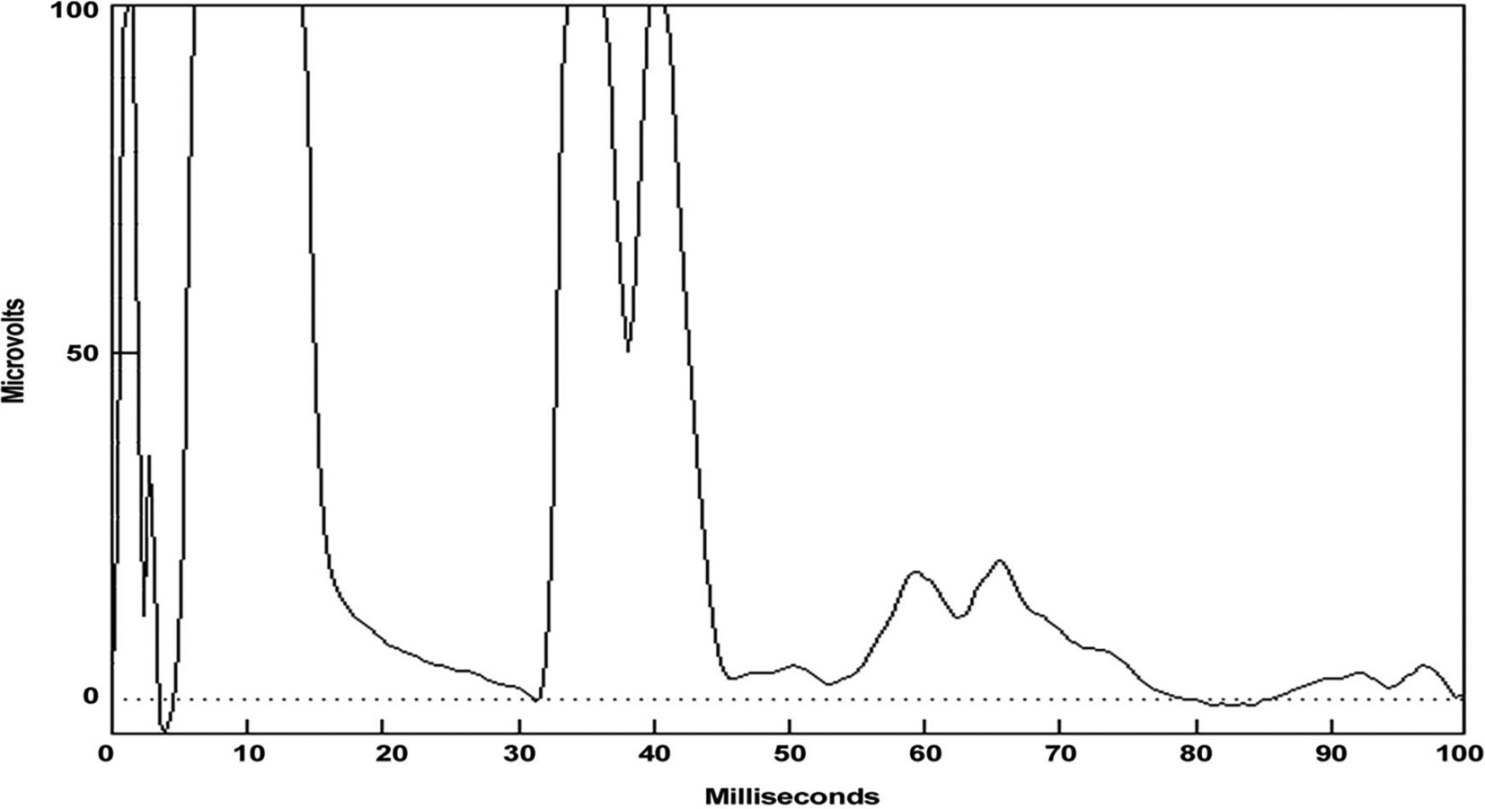
Enhanced Abductor Pollicis Brevis long latency electromyographic (EMG) reflexes at rest to median nerve stimulation at 53 msec and a smaller wave at 84 msec. This demonstrates an enhance transcortical reflex response.

Such an EMG response demonstrates sensorimotor hyperexcitability (Shibasaki, 2000). In the clinical setting of myoclonus, this is evidence of a cortical origin. These myoclonus patients commonly have myoclonus after stimulation (reflex myoclonus). Reflex myoclonus may be clinically elicited by touch or muscle stretch. For upper extremities, briskly abducting the thumb may evoke a reflex jerk. This reflex jerk can be confirmed with EEG-EMG polygraphy, but it is easier to prove reflex myoclonus by testing for long latency EMG responses to electrical nerve stimulation. A consistent gross apparent EEG transient may come before the myoclonus EMG discharge with each stimulus. For the thumb, stimulation of the median nerve stimulation can show EMG discharges at 50 msec latency or greater (range 40–60 msec) from the stimulus. At intervals of 20–40 msec, more than one EMG discharge may be seen (Fig. 5). In Fig. 5, the rectified long-latency EMG reflexes have onset at approximately 55 msec and 85 msec after electrical stimulation at the median nerve. No response should be present at rest in a normal patient. It is important to note that there must be proper relaxation must be obtained, or a possibility of a false positive response could occur.

d) Enlarged cortical somatosensory evoked potentials (SEPs)

**Fig. 6:**
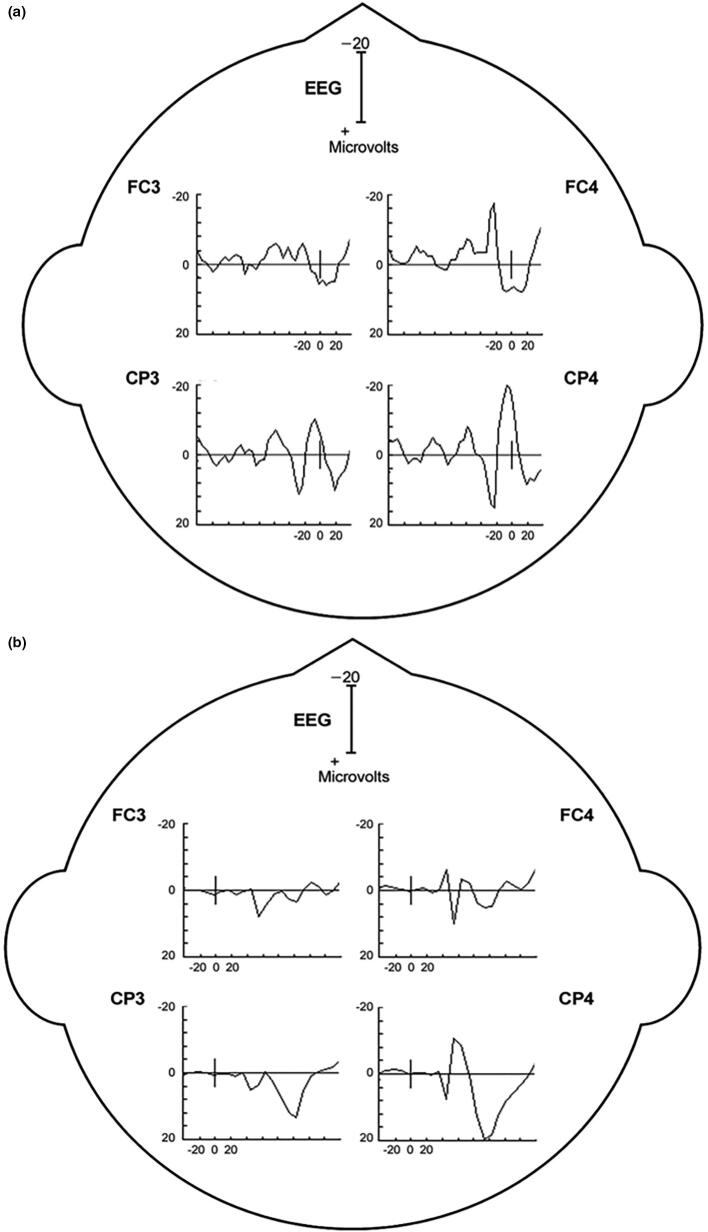
A: Back-averaging of a focal cortical transient preceding averaged left arm myoclonus electromyographic (EMG) discharges in a patient with cortical myoclonus. B: Enlarged cortical somatosensory-evoked potentials (SEP) from the same patient. Note the similarity of the P25-N33 wave dipole in both A and B. In both A and B, there is a positive wave in the CP4 electrode with a simultaneous FC4 negative wave. Averaged ear reference was used.

For myoclonus reflex sensitivity documentation in cortical myoclonus, enlargement of the cortical SEP parietal wave complex is important corroboration for the cortical reflex physiology (Shibasaki, 2000). An important property of the enlarged cortical SEP P25-N33 wave is that it possesses similar morphology and topography to the EEG transient derived from the EEG-EMG back-averaging (Fig. 6). Moreover, the interval between the P25 peak to onset of any recorded long latency EMG response is close to the latency between the back-averaged EEG transient positive wave deflection to the onset of the myoclonus EMG discharge. Enlarged or even “giant” SEPs are a very common finding in Sialidosis type 1 patients with myoclonus, and it has been suggested that such a finding be considered a useful marker for this syndrome (Fan et al., 2020). In locations where EEG-EMG polygraphy is difficult to obtain or not available, enlarged SEPs could be useful to corroborate cortical myoclonus. However, caution is warranted since enlarged SEPs and cortical myoclonus are not uniformly associated.

e) Elevated cortico-muscular coherence

Coherence is defined as the degree of fixed relationship between the phases of two oscillating waves at a given frequency. Thus, cortico-muscular coherence is a frequency domain measure of correlation degree between EEG (at a particular electrode location) and EMG signal of a specific muscle. This can provide information about the relationship between cortical EEG signals and muscle activity in myoclonus patients. Brown et al. have found exaggerated EEG-EMG and EMG-EMG coherence patterns for subjects with myoclonus (Brown et al., 1999, Grosse et al., 2003, van Rootselaar et al., 2006). They have suggested that this abnormality may have potential diagnostic value for supporting cortical myoclonus physiology. Moreover, elevated coherence may be more sensitive than EEG-EMG back-averaging. This has been suggested particularly for relative high frequency myoclonus EMG discharges, such as those around 10 Hz or higher (Grosse et al., 2003). In another study, elevated cortico-muscular coherence was found in the small distal myoclonus of Parkinson’s disease (Caviness et al., 2003). This suggests that motor cortical rhythms are pathologically coupled to motor neurons in some cases of Parkinson’s disease. These authors also found that cortico-muscular coherence is elevated even when myoclonus is not occurring and that it elevates further around the time of myoclonus.

f) Focal electrographic seizure discharges

When cortical myoclonus arises in a sudden and paroxysmal manner, it is commonly termed, “partial epilepsy with motor symptomatology.” Nevertheless, the basic movement disorder phenotype is classified as focal myoclonus, either occurring as paroxysms of repetitive focal jerks, or as *epilepsia partialis continua* when occurring for long periods. Negative myoclonus can also occur (Yang et al., 2020). There are various ictal EEG discharges that can be seen in the appropriate contralateral motor area during a focal motor seizure. Repetitive focal spike, spike and wave, sharp wave, rhythmic theta or delta activity, or desynchronization may occur. In some cases, no grossly observable EEG activity is seen, and back-averaging may uncover a transient in some patients. In the case of *epilepsia partialis continua*, the above-mentioned transients may show periodicity and occur with the pattern of periodic lateralizing epileptiform discharges (PLEDS). Despite focal EEG discharges or clinical onset, generalized spread can occur resulting in generalized convulsion.

##### 1.2.2.4 Cortical myoclonus criteria

Electrographic seizure on EEG (f), or gross EEG pre-myoclonus discharges (b) constitute a definitive demonstration for cortical origin myoclonus physiology. Brief surface myoclonus EMG discharges with a demonstrated EEG-EMG back-averaged EEG transient (b) is also confirmatory for cortical myoclonus. Enhanced long latency EMG reflexes (c) and/or enlarged cortical SEP (d) are supportive of cortical myoclonus. It is the exaggerated reflex features of enhanced long-latency EMG reflexes to nerve stimulation (c) and/or enlarged cortical SEP components (d) that are typical of the classification of “cortical reflex myoclonus.” In the setting of clinical myoclonus, elevated cortico-muscular coherence (e) supports a cortical origin for myoclonus. However, criteria using cortico-muscular coherence should be based on laboratory established cortical myoclonus and suitable control values. Although these criteria are commonly used, they are mainly based on smaller case series and expert opinion. They need further validation as well as consensus (van der Veen et al., 2021).

##### 1.2.2.5 Relationship of cortical myoclonus to epilepsy

The significance of cortical myoclonus and its relationship to epilepsy needs consideration and comment. The establishment of a cortical physiology for myoclonus implies abnormal excitability in the cortex, which is also involved in the pathophysiology seizure disorders in general. Indeed, much attention has been given to the relationship between cortical myoclonus and epilepsy, given the presence of a presumed hyperexcitability of sensorimotor cortex in both instances. The classic paper by Obeso et al. examined the overlap of clinical and neurophysiological findings in patients with cortical myoclonus (Obeso et al., 1985). These patients, when taken together, exhibited a “spectrum” of varying combinations of action and stimulus-sensitive myoclonus, spontaneous myoclonus, *epilepsia partialis continua*, focal motor seizures, and secondary generalized convulsions. This paper posited that patients with these varied manifestations exhibited points along this spectrum with differences in abnormal sensorimotor cortex neurophysiology. Hallett has divided myoclonus into epileptic and non-epileptic (Hallett, 1985). He hypothesized that cortical reflex myoclonus is a fragment of partial epilepsy, reticular reflex myoclonus is a fragment of generalized epilepsy, and primary generalized epileptic myoclonus is a fragment of primary generalized epilepsy. The fact that anti-seizure medication represents the best available treatments for cortical myoclonus means that more exploration of this complex relationship is needed.

#### 1.2.3 Cortical-subcortical myoclonus

The classification category “cortical-subcortical” myoclonus refers to myoclonus arising from abnormal hyperexcitable interaction in both cortical and subcortical areas. Clinically, myoclonic seizures are the prime myoclonus example that arises from this physiology. Indeed, evidence from imaging and animal models suggest that some generalized seizure phenomena arise from paroxysmal bidirectional oscillation in cortical-subcortical areas (Deppe et al., 2008, Savic et al., 1994, Snead, 1995). Thus, the abnormal input of subcortical input plays a significant role in this myoclonus physiology. Despite the subcortical involvement, the cortical discharge precedes and drives the myoclonus jerk. This myoclonus usually occurs in paroxysms from rest and can be associated with other seizure types. The myoclonus is usually prominent in limbs and bilateral/generalized, but other distributions also occur (Oguni et al., 1994). The type of abnormality that gives rise to such bidirectional over-excitation between cortical and subcortical areas likely arises from intrinsic electrical abnormalities at the neuronal level. Thus, it is understandable that genetic mutations relating to ion channels and ion buffering have been associated with various myoclonic epilepsy syndromes (Bradley et al., 2008). It is interesting to note that both myoclonic seizures and cortical myoclonus can occur in FCMTE and sometimes in progressive myoclonic epilepsy (PME) syndromes (Lasek-Bal et al., 2019, Licchetta et al., 2013, van Rootselaar et al., 2002).

**Fig. 7:**
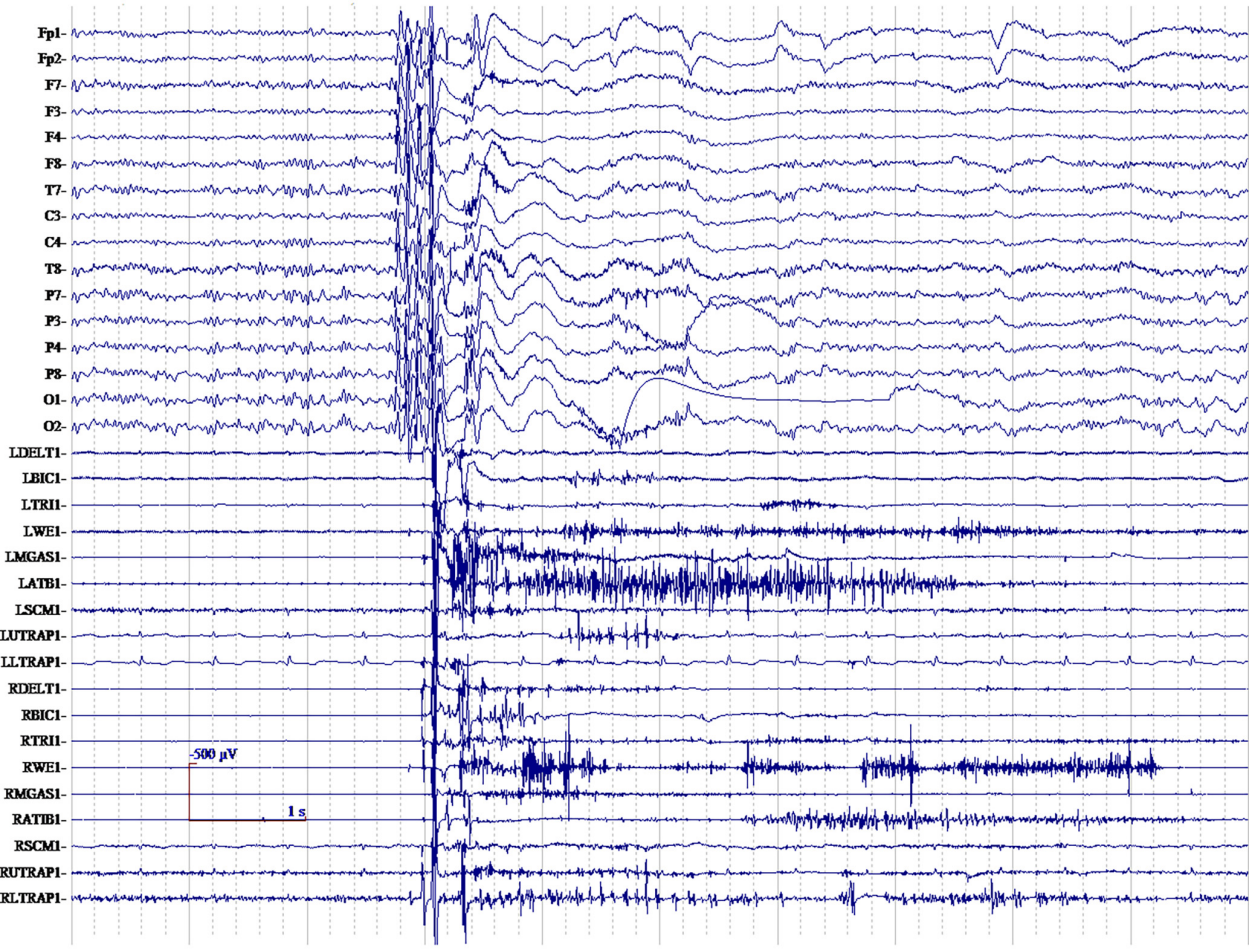
Generalized myoclonus electromyographic (EMG) discharges with a typical cortical-subcortical physiology is shown with polyspike encephalographic (EEG) discharges occurring with the myoclonus. Also in this patient, there were common interictal spike and wave EEG discharges without myoclonus. These cortical-subcortical electrophysiology findings should be noted as a strike contrast to cortical myoclonus, despite the fact that EEG discharges are present in both.

The electrophysiological hallmark of cortical-subcortical myoclonus is the generalized spike and wave EEG discharge (Fig. 7). The myoclonus EMG discharge duration is usually less than 100 ms. Clinical myoclonus grossly correlates with the EEG discharge, but commonly there is jitter in the interval between EEG discharge and myoclonus. In particular, the polyspike trains will correlate with single or multiple jerks. There is a generalized EEG distribution, often predominantly fronto-central occurring up to 20 Hz (Serafini et al., 2013). Pure generalized spike and wave discharges commonly occur without myoclonus as interictal phenomena. Polyminimyoclonus of central origin is an example of the cortical-subcortical physiology, in which jerk-locked back-averaging shows a slow bilateral generalized negativity. It has been described as a fragmentation of generalized epilepsy and called primary generalized epileptic myoclonus (Wilkins et al., 1985). In Fig. 7, generalized myoclonus with a typical cortical-subcortical physiology is shown with polyspike EEG discharges occurring with the myoclonus. Also in this patient, there were common interictal spike and wave EEG discharges without myoclonus. These cortical-subcortical electrophysiology findings should be noted as a strike contrast to cortical myoclonus, despite the fact that EEG discharges are present in both myoclonus physiology types. As they are so closely related not all clinical classification schemes include cortical-subcortical myoclonus as a separate entity, but put this form partly (jerks) with the cortical myoclonus or classify it as a form of epilepsy (Zutt et al., 2018, Zutt et al., 2015).

The EEG discharge frequency (both ictal and interictal) varies across the different syndromes and is somewhat characteristic of the syndrome (Hirano et al., 2009). The 4–6 Hz spike or polyspike and wave generalized discharge is typical for juvenile myoclonic epilepsy, and photosensitivity is a common associated feature. Myoclonus is associated with absence seizures in up to 45 % of cases (Penry et al., 1975). Most of the time, the myoclonus in the absence seizure is located in the eyelid, other facial or midline muscles, or less commonly in the limbs. The myoclonus is usually timed to the generalized spike and wave discharges on the EEG. In classic typical absence, the frequency of the spike and wave discharges is 2.5–4 Hz and this is only an ictal pattern. In atypical absence, as seen in Lennox-Gastaut syndrome, 1–2.5 Hz occurs and may be seen as an ictal or interictal pattern.

#### 1.2.4 Subcortical/non-segmental myoclonus

Characterization of the physiology or anatomy of subcortical myoclonus poses challenges. Unlike cortical myoclonus, there is seldom a clear definition of the generating physiology or anatomy, e.g., EEG transient. This uncertainty creates a barrier to confident classification of specific cases as subcortical physiology from electrophysiological findings alone. Rather, the clinical circumstances and lack of findings from other physiology types, can be used to suggest subcortical physiology for the clinical myoclonus. However, the uncertainty remains for using this myoclonus physiology classification category in practice.

The clinical and neurophysiological characteristics of subcortical myoclonus are more variable than for those in cortical or cortical-subcortical myoclonus. This likely stems from the relatively more disparate subcortical centers that can be associated with myoclonus. The anatomical locations that are posited to dysfunction within this category extend from the basal ganglia to the spinal cord. However, in all examples, the source transmits its excitatory influence to muscle segments far beyond its location, i.e., non-segmental. The myoclonus EMG duration observed has a wide range of 25–300 ms. The temporal relationship between the agonists and antagonists muscle activation is also variable.

**Fig. 8:**
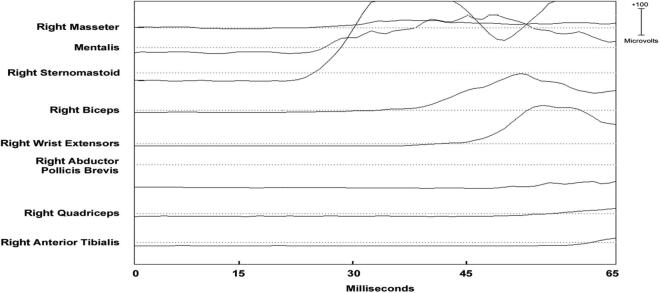
Expanded time scale that shows a simultaneous ascending and descending order of recruitment from a presumed lower brainstem source. Note that the sternomastoid muscle is recruited first. The electromyographic (EMG) discharges are rectified and averaged from 20 trials. Time zero marks the time that the examiner touched the shoulder to elicit the generalized jerks.

Two types of EMG discharge recruitment pattern are generally observed. First, subcortical/non-segmental myoclonus can demonstrate simultaneous rostral and caudal recruitment of muscle segments along the neuraxis from a localized source. The classic example is reticular reflex myoclonus (Fig. 8). This myoclonus pattern shows rostral and caudal recruitment of muscle segments along the neuraxis from a localized source. This excitation elicits jerks that may be generalized or bilateral and widespread. Often, this myoclonus is reflex sensitive. The EMG duration may range from 25 to 300 ms, and muscles in the same segment show nearly synchronous activation. The simultaneous rostral and caudal recruitment order is the characteristic finding of the surface EMG polygraphy. The rostral and caudal spread occurs more slowly than what is observed in the corticospinal pathways seen in cortical myoclonus. Fig. 8 demonstrates these properties of reticular reflex myoclonus. If any EEG activity is observed, it is seen after the first muscle is activated and is not time-locked in a meaningful way to the EMG activation. In reticular reflex myoclonus, the myoclonus source is thought to be in the lower brainstem reticular formation (Hallett et al., 1977). One should be aware that there are only very few cases described with reticular myoclonus (Beudel et al., 2014, Hallett et al., 1977). Brainstem motor systems are particularly involved in axial and bilateral movements and are tightly linked to subcortical reflex centers. Thus, brainstem myoclonus is often generalized, especially axial, and stimulus sensitive.

**Fig. 9:**
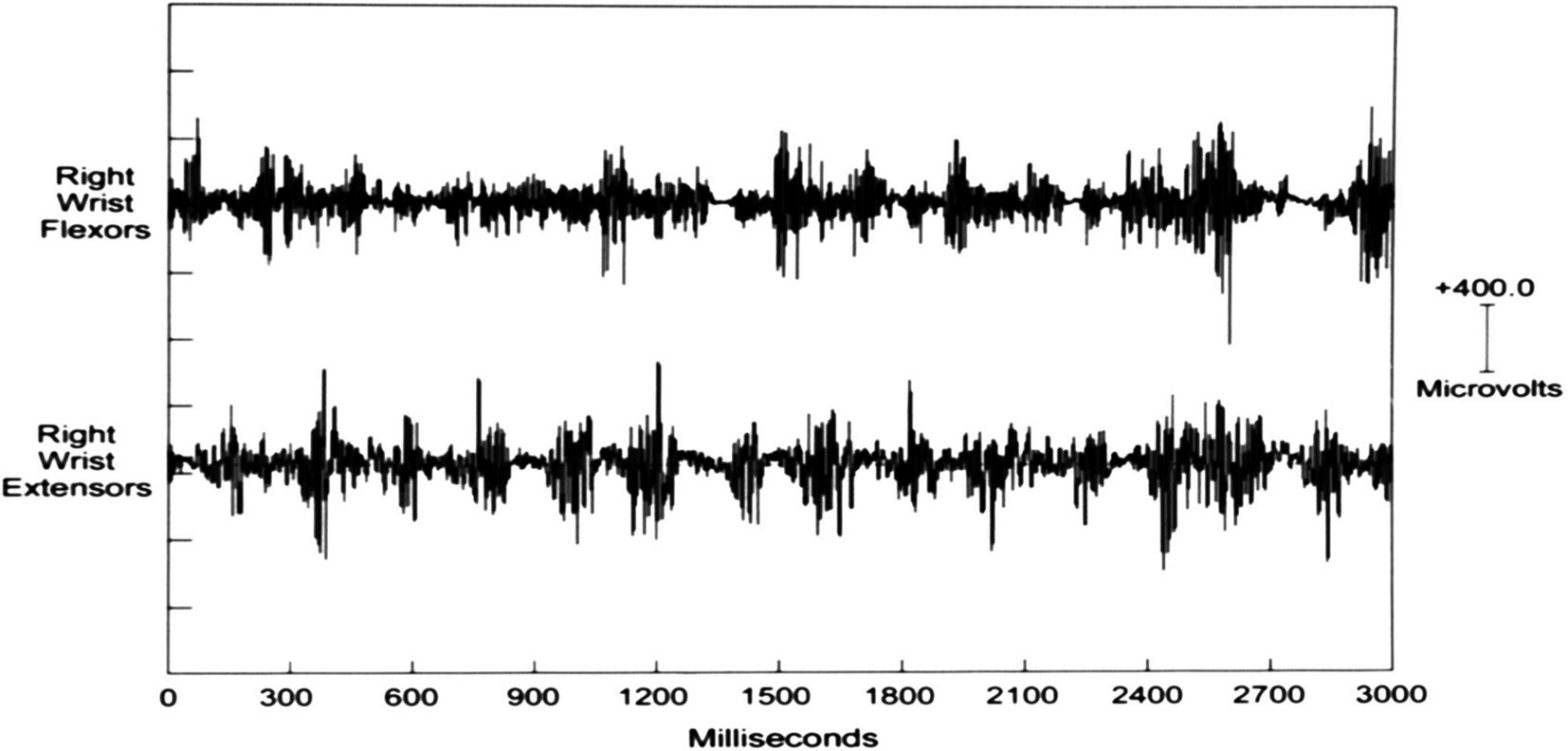
Right wrist multichannel surface electromyographic (EMG) recording from a patient with myoclonus –dystonia syndrome during postural activation eliciting the right arm myoclonus. Note the long duration of the shortest EMG discharges (100–200 ms) in addition to longer duration discharges. The myoclonus EMG discharges are irregular with respect to amplitude, duration, and timing between agonist and antagonist muscles.

The second general type of EMG discharge recruitment pattern seen in subcortical/non-segmental myoclonus is multifocal, most common during muscle activation. Although this pattern is also typical for cortical myoclonus, the EMG discharge duration seen here is longer and much more variable. Reflex features are not common. Other electrophysiological features seen can reflect other movement disorders seen in that syndrome or etiology. Classic examples are the opsoclonus-myoclonus syndrome and the myoclonus-dystonia syndrome, previously essential myoclonus. In the case of myoclonus caused by autosomal dominant ε-sarcoglycan gene mutation as the etiology, characteristic features include upper extremity and trunk/neck involvement with notable worsening with action, onset before age 20 years with a fairly benign course, absence of other severe neurologic deficits, and normal EEG. Other genotypes have been described (Jones et al., 2019). Alcohol responsiveness is common enough to be characteristic. There is dystonia of a similar distribution in many cases. The clinical-electrophysiology characteristics of those patients with mutations in the ε-sarcoglycan gene have been classically described by (Li et al., 2008, Roze et al., 2008). Their series showed an average EMG duration of 95 ms, range 25–256 ms. No findings of cortical hyperexcitability were found, including a lack of back-averaged cortical potentials time-locked to the myoclonus. One study linked possible oscillatory activity in the globus pallidus internus to this syndrome (Foncke et al., 2007). Subcortical regions highly express ε-sarcoglycan, and a dysfunctional widespread subcortical network has been proposed (Chan et al., 2005, Menozzi et al., 2019). Fig. 9 shows right wrist surface EMG during postural muscle activation in a myoclonus-dystonia syndrome patient with an ε-sarcoglycan gene mutation.

##### 1.2.4.1 Orthostatic myoclonus

**Fig. 10:**
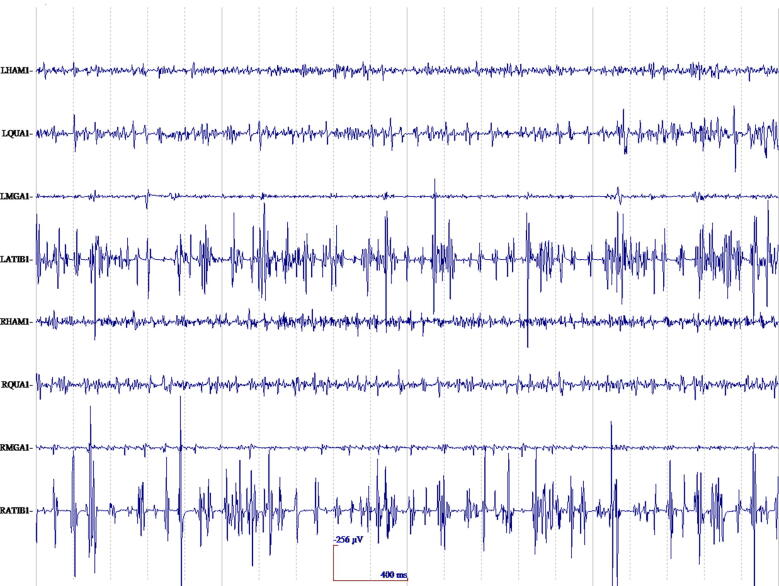
Multichannel surface electromyographic (EMG) recording from the face of a patient with hemifacial spasm during a train of right facial myoclonic jerks and their corresponding EMG discharges. Similar discharges are seen across all facial (CN VII) nerve innervated muscles. EMG discharge duration variability with the facial myoclonus is demonstrated. This patient also had much longer EMG discharges associated with sustained spasms (not shown).

Orthostatic myoclonus was described in 2007 in elderly patients with gait problems (Glass et al., 2007). It has clinical and electrophysiological distinction from both classic and slow orthostatic tremor (Rigby et al., 2015). The EMG discharges have a highly variable duration from 25 to 100 ms while showing both synchronous and asynchronous discharge relationships between muscles, giving a disorganized appearance to the EMG polygraphy (van Gerpen, 2014, Gunduz et al., 2018) (Fig. 10). Semi-rhythmic discharges between 4 and 11 Hz occur (van Gerpen, 2014). Both positive and negative myoclonus are seen. There has been no back-averaged EEG transient reported. This finding and the lack of exaggerated cortical reflex findings has led to some authors proposing the neurophysiology as subcortical. However, more study is needed, and technical factors may make it difficult to determine.

#### 1.2.5 Segmental myoclonus

Segmental myoclonus is defined as rhythmic or semi-rhythmic involuntary contractions of muscle groups supplied by one or more *contiguous* segments of the brainstem and/or spinal cord (Caviness, 2012). This movement is usually caused by abnormal segmental physiology within interneuron pools that can influence movement. The movements are overall rhythmic and usually very persistent. Insensitivity to sensory stimuli and to cognitive state is characteristic. A number of different distributions may be seen in segmental myoclonus, including palatal, spinal, brachial, oculofacialmasticatory myorhythmia, abdominal/truncal, and even diaphragmatic. Palatal segmental myoclonus is the most common, although recent literature also detected a lot of functional cases amongst essential palatal myoclonus (Vial et al., 2020). The diagnosis of segmental myoclonus is important since it can be caused by a brainstem or spinal cord pathology. An MRI of the appropriate segment(s) should be obtained to investigate for the presence of pathology. Treatment of segmental myoclonus usually leads to only incomplete suppression of the movements. It is rarely possible to treat the underlying etiology of segmental myoclonus, and controlled studies for symptomatic treatment are lacking. Besides medication trials based on anecdotal evidence, botulinum toxin injections have had some success. However, such botulinum toxin therapy should be discussed with and done by experts.

##### 1.2.5.1 Palatal segmental myoclonus

The repetitive movements of palatal myoclonus are most commonly within the 1–4 Hz range. In some cases, the movement flow is sinusoidal, while in other cases it has a jerk or jerk-like character. Clinically, palatal myoclonus cases are divided into “essential” (EPM) and “symptomatic” (SPM) categories with described properties. In EPM, there is usually activation of the *tensor veli palatini* muscle without the activation of other muscles. An identifiable MRI lesion has never been found. In SPM, there is usually activation of the *levator veli palatini* muscles, but involvement of other muscles is common. MRI may show a lesion in the brainstem somewhere in the Guillain-Mollaret triangle. Commonly, palatal myoclonus appears very rhythmic and sinusoidal in which case experts will use the term tremor rather than myoclonus. There are reported electrophysiological findings that are variable and may even be indirectly associated. For example, brainstem auditory evoked potentials have had abnormal findings in some individuals with palatal myoclonus (Westmoreland et al., 1983). There are normal EEG and SEP. Deuschl pointed out differences in electrophysiological testing between EPM and SPM (Deuschl et al., 1994). Blink reflex activity, jaw jerk, and masseteric silent period had only polysynaptic brainstem reflex abnormalities in EPM (which in retrospect might have been false positive findings), whereas SPM patients can have abnormalities of monosynaptic, oligosynaptic, and polysynaptic brainstem reflexes.

Cases of EPM have obnoxious clicking often as a prominent symptom. Recent literature also detected many functional cases amongst EPM (Vial et al., 2020) and it is uncertain whether there is any other etiology. Botulinum toxin has been used with some success in these cases. Another example of clicking coming from segmental myoclonus is middle ear muscle myoclonus, affecting the stapedius and/or tensor tymphani muscles (Hutz et al., 2021) although it is likely that many of these cases are also functional.

##### 1.2.5.2 Spinal segmental myoclonus

In spinal regions, the surface EMG shows rhythmic or semi-rhythmic discharges in muscles supplied by the affected spinal segment. The affected muscles usually show nearly synchronous or a phase-locked activation in the EMG polygraphy. The common frequency range is 1–3 Hz with a broad reported range of 0.2 to 8 Hz. Surface EMG discharge duration varies from case to case between 50 and 500 ms (Bagnato et al., 2001, Calancie, 2006, Keswani et al., 2002). This form of myoclonus is extremely rare. Typically, these discharges are continuous and are not affected by reflex stimuli (Warren et al., 2003). There are normal EEG and SEP. Similar to palatal myoclonus, there are reported, and again perhaps indirect abnormalities in evoked potential studies. Somatosensory evoked spinal potential recovery curves were abnormal in a case that involved lumbar myotomes. It was suggested that dorsal horn interneurons are abnormally hyperactive and may contribute to the motor neuron excitability (Di Lazzaro et al., 1996).

##### 1.2.5.3 Spinal propriospinal myoclonus

Patients with propriospinal myoclonus clinically have jerks with trunk flexion or extension with phasic axial muscle activation (Brown et al., 1991d). Proximal limb muscles may occur in the jerk bilaterally, but the dominant action is in the axial muscles. These jerks occur from rest and/or activated by stimuli such as touch, deep tendon reflex, or muscle stretch. The EMG discharge lasts from 50 to 300 msec, or rarely longer. Both reciprocal and co-contracting agonist–antagonist relationships have been observed. The EMG activation pattern is a simultaneous bilateral rostral and caudal recruitment originating from the spinal cord origin. The activation speed of consecutive muscles is slower than for the corticospinal (pyramidal) pathway and is thought to be propriospinal. It is important to notice that currently the opinion is that most patients with propriospinal myoclonus are considered functional. The muscle activation pattern in these patients can be stereotyped but preceded by a Bereitschaftspotential in many cases (Erro et al., 2013, van der Salm et al., 2014).

#### 1.2.6 Neurodegenerative and infectious disorders with characteristic electrophysiological findings

There are certain well-known syndromes and disorders, such as Alzheimer’s disease, Creutzfeldt-Jakob disease, subacute sclerosing panencephalitis, Lewy Body dementia, corticobasal degeneration, multiple system atrophy, and progressive supranuclear palsy which have myoclonus electrophysiological findings that are considered characteristic. The precise physiology and anatomy of the myoclonus is not always known but the findings can be found in Table 1.

**Table 1:** Characteristic electrophysiological findings of neurodegenerative and infectious disorders that can presenting with myoclonus.

Disorder	Prevalence of myoclonus	Surface electromyography	Electro-encephalography	Additional investigations
Alzheimer’s disease	Common	Different presentation of myoclonus across different cases and disease stages: 1) Multifocal myoclonus, with brief discharges in action and rest is most common. 2) Polyminimyoclonus, small, multifocal twitched in fingers and hands. 3) Rapid progression with rest and generalized myoclonus with < 100 ms discharges, agonist-only pattern or with cocontraction with other muscles.	Progressive decrease in background rhythm and increased slower frequencies. 1) Variable JLBA with focal contralateral central negativity in EEG with onset 20–40 ms pre-myoclonus and 40–80 ms duration. 2) JLBA with bifrontal EEG negativity with onset 50–170 ms pre-myoclonus and 100–180 ms duration. 3) Periodic sharp waves similar to CJD have been described.	Enlarged SEP can be found. Variable presence of long latency responses to median nerve stimulation.

Creutzfeldt-Jakob disease	Common	Focal, multifocal, bilateral, or generalized distribution. Discharges around 60 ms. Negative myoclonus can be present.	Periodic synchronous discharge time-locked to myoclonus with 100–160 ms duration and latency of 50–85 ms to myoclonus.	Enlarged SEP can be found.

Subacute Sclerosing Panencephalitis	Unknown	Quasiperiodic jerks with sustained ‘dystonic’ posture lasting up to approx. 1 s in upper extremities. Burst duration > 200 ms. Fairly resistant to stimuli.	Associated to generalized complex EEG discharges: high voltage (300–1500 μV), repetitive, polyphasic and sharp and slow wave complexes with 500–2000 ms in duration, occurring every 4–15 s.	–

Lewy Body dementia	15–25 %	Multifocal or generalized myoclonus during action and rest. Larger amplitude compared to myoclonus in Parkinson’s disease with duration of 20–40 ms.	Focal short latency EEG transient prior to EMG discharge.	–

Corticobasal degeneration	Unknown	Synchronous rhythmic repetitive trains of 25–50 ms discharges in agonist–antagonist pairs.	EMG-EEG transient is usually elusive. Cortical correlate back-averaged from magneto-encephalography.	SEP is unremarkable or altered without enlargement. Enhanced long latency EMG reflexes. Short C-reflex

Multiple System Atrophy (MSA)	Most studied in MSA cerebellar type.	Polyminimyoclonus during postural activation with duration < 100 ms or action myoclonus. Somatosensory stimulus-sensitive jerks.	No back-averaged cortical correlate in few patients with polyminimyoclonus, but EEG transient prior to EMG discharge was present in patients with action myoclonus.	Enlarged SEP can be seen. Enhanced long latency EMG responses.

Progressive supranuclear palsy	Rare	Action myoclonus with discharges < 50 ms.	Gross correlation between EEG epileptiform activity and EMG discharges.	–

CJD, Creutzfeldt-Jakob disease; EEG, electro-encephalography; EMG, electromyography; JLBA, jerk-locked back-averaging; SEP, sensory evoked potential.

#### 1.2.7 Functional jerks

Jerks arising from a functional mechanism may appear brief and thus able fit with a myoclonus phenotype. However, it is often observed that functional jerks appear more complex and variable than a non-functional myoclonus phenotype. Properties of functional jerks have been categorized: (1) inconsistent character of the movements (amplitude, frequency, and distribution) and other features incongruous with typical “non-functional” myoclonus, (2) marked reduction of the myoclonus with distraction, (3) spontaneous periods of remission, and (4) acute onset and sudden resolution (Zutt et al., 2019). Although clinically useful, these properties are subjectively assessed. Thus, clinicians, patients and families may want more objective “proof”. Electrophysiology testing may offer the clinician some assistance with evaluating these patients, but one must exercise caution when interpreting the results. Such testing should be considered an adjunct to clinical evaluation and not a substitution for clinical diagnosis.

Longer EMG discharges are possible in functional myoclonus and the discharge pattern may have a non-stereotyped appearance from jerk to jerk. Stimulus-evoked jerks or jumps with a mean latency in excess of 100 msec and variability of the latency suggest voluntary or functional jerks (van der Salm et al., 2012).

The presence of cortical myoclonus electrophysiology findings should, of course, be taken as evidence against those jerks being functional. High amplitude EMG discharges < 50 ms duration that correlate with a moderate amplitude jerk strongly favors involuntary myoclonus for that particular type of movement. Enlarged cortical SEP, C-reflexes, back-averaging of a short duration (<100 ms) EEG wave preceding the jerk suggest non-functional myoclonus.

**Fig. 11:**
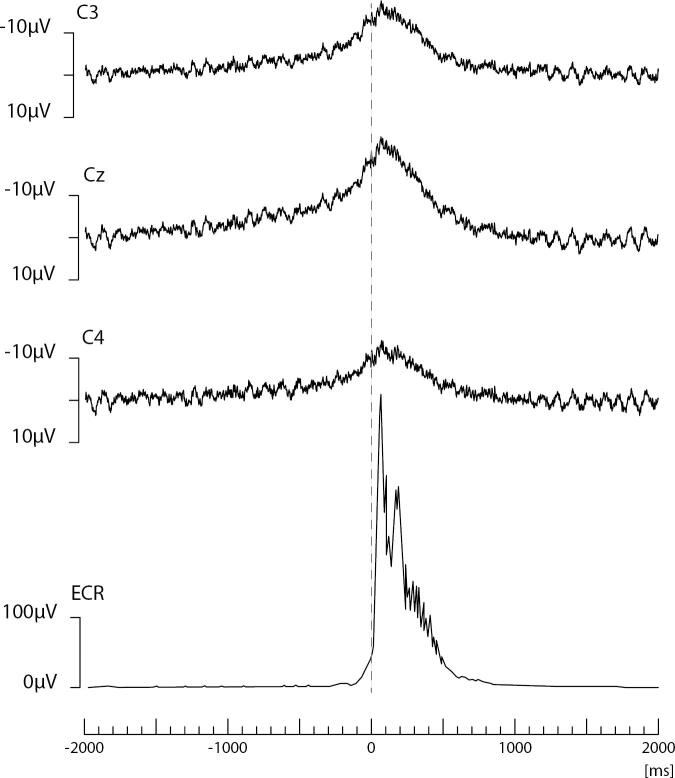
Representative recording of an individual case with jerky movements in the arm of functional origin. Four seconds of raw electroencephalographic (EEG) and electromyographic (EMG) data of the extensor carpi radialis (ECR). Note the long duration EMG bursts (+/- 500 ms). After back-averaging of 70 epochs of jerks, a Bereitschaftspotential can be seen be on C3, Cz and C4, which starts approximately 1 s before jerk onset.

The Bereitschaftspotential (BP) is a back-averaged negative “slow” EEG cortical potential that occurs before self-paced, voluntary phasic movements (Fig. 11). The first phase is relatively widespread with a vertex prominence, slowly increasing from approximately 2000–1000 msec to approximately 650–450 ms before voluntary movement. Then, in the second phase, the negativity rises faster and lateralization occurs before the voluntary movement. The presence of a BP preceding functional myoclonus can be supportive evidence for a functional etiology (van der Salm et al., 2012). Event-related desynchronization (ERD) in the beta band has also been used to suggest a functional etiology for a jerk. There is evidence to propose the combined use of beta band ERD and quantitative BP measures in suspected functional jerk patients (Beudel et al., 2018).

#### 1.2.8 Peripheral myoclonus

**Fig. 12:**
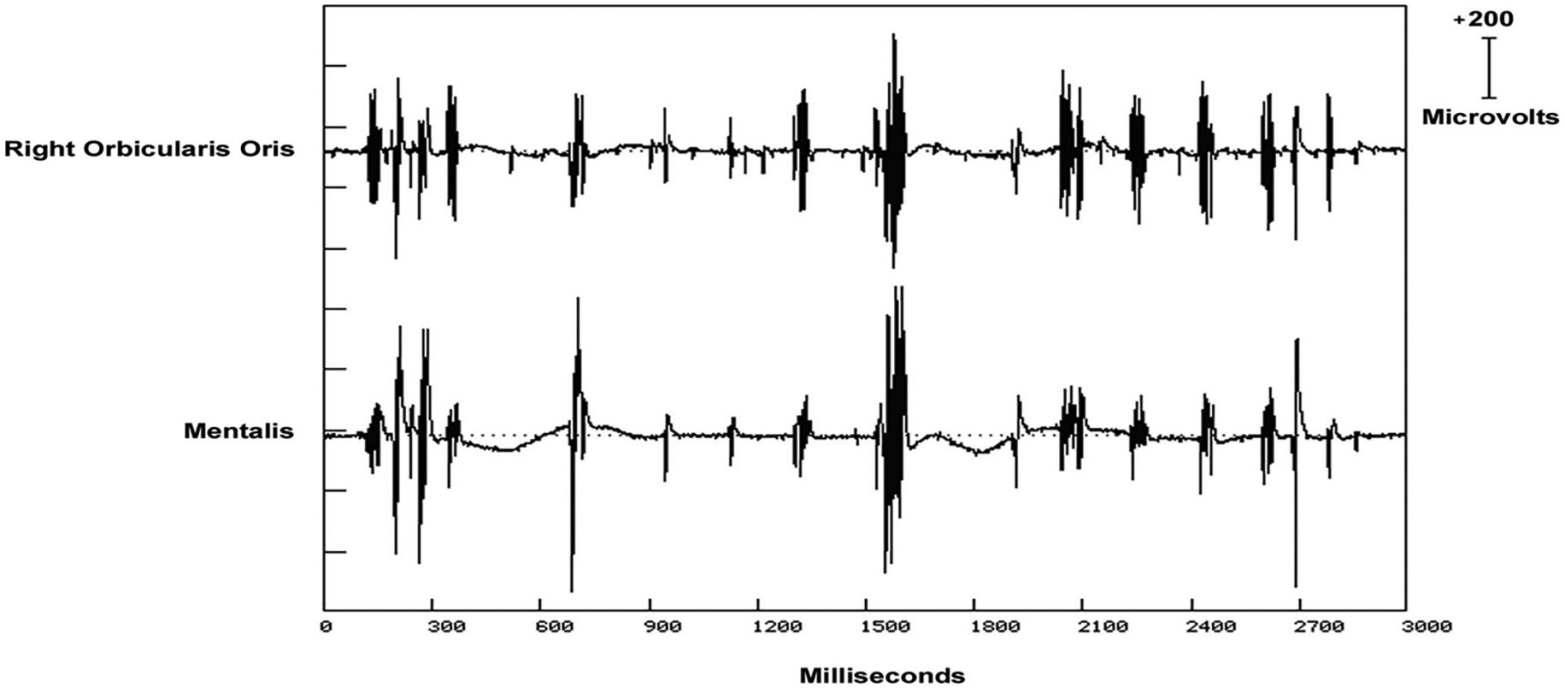
The electromyographic (EMG) discharges have a highly variable duration from 25 to 100 ms while showing both synchronous and asynchronous discharge relationships between muscles, giving a disorganized appearance to the EMG polygraphy recording. This irregular pattern correlates with lower extremity myoclonus and thus “orthostatic myoclonus”.

It is believed that peripheral nerve abnormality may produce myoclonus (Valls-Solé, 2007). The most common example is the hemifacial spasm (Jankovic and Pardo, 1986). Even though sustained spasms may dominate the clinical picture for hemifacial spasm patients, very brief movements are often present as well. It is these movements that can be considered as peripheral myoclonus. Even so, there is controversy with how the term is used. For example, some studies report myoclonus associated with peripheral nervous system lesions, but propose that the myoclonus results from “central reorganization” (Tyvaert et al., 2009). There is often marked variability in duration from EMG discharge to discharge from 50 to 150 ms. The EMG discharges supplied by the same nerve are nearly synchronous. Fig. 12 show these findings from a patient with facial jerks in association with hemifacial spasm.

### 1.3 Discussion and summary

Electrophysiological studies can be used to: 1) confirm myoclonus as the accurate movement disorder phenotype, 2) provide neurophysiological classification and localization information, and 3) in some instances, provide some degrees of diagnostic specificity. It should be realized that a single disease or patient may have more than one myoclonus neurophysiology type. In a study by Zutt et al., it was found that electrophysiological testing in the context of clinical findings altered the myoclonus diagnosis and the subtype in 53 % of patients (Zutt et al., 2018). The importance of electrophysiological testing in myoclonus is greatly enhanced by the fact that clinicians may use neurophysiological classification to develop treatment strategy. In summary, it is both indicated and critical for electrophysiological studies and neurophysiology classification be performed on myoclonus patients.

## 2 Tics

### 2.1 Introduction

Tics belong to the spectrum of hyperkinetic movement disorders. Tics have a wide range of etiologies, but are most commonly encountered within the spectrum of primary tic disorders, including Tourette syndrome (TS) with prevalence rates of up to 1 % in pediatric populations (Ganos and Martino, 2015). The diagnosis of TS can be made when two or more motor tics and at least one vocal tic have been present in an individual’s life before the age of 18 for over a year (American Psychiatric Association, 2013). In the presence of motor or vocal tics only, the diagnosis of a chronic motor/vocal tic disorder is made. Finally, a diagnostic entity within primary tic disorders is that of provisional tic disorder (Kim et al., 2019), denotes the presence of tics in children and adolescents, when this has been documented for less than a year. Beyond primary tic disorders, tic behaviors have been described in a range of other neurodevelopmental, neurometabolic and neurodegenerative disorders, including neurogenetic disorders (see (Ganos and Martino, 2015) for review). For example, tics are a common finding in people with autism spectrum disorder, and in individuals with monogenetic conditions presenting with features from the autism spectrum, such as fragile-x syndrome and (late-treated) phenylketonuria (Ganos and Martino, 2015, Mainka et al., 2021). Tics are also common in several choreatic syndromes, including Huntington’s disease and chorea-acanthocytosis. Although a discussion of the different syndromic associations is not the topic of this chapter, it is important to document that a closer examination of the different tic etiologies could provide very specific clues as to possible risk factors for tic emergence. Of note, tic phenomena have also been described in functional movement disorders, with an increase in prevalence over the past few years (Demartini et al., 2014, Ganos et al., 2019, Ganos et al., 2016, Heyman et al., 2021). However, the presentation of many of these phenomena is often different than in tics documented in primary tic disorders, and hence their pathophysiological underpinnings could also differ.

### 2.2 Clinical features of tics in primary tic disorders

Tics are brief, discreet movements or sounds that share most of the phenomenological characteristics of volitional behavior, but occur repetitively, in irregular time intervals and are not embedded to a discernible context. Tics can occupy the entire range of possible behavioral output of different effector muscles, and indeed any possible movement can also be part of a tic behavior. Importantly, tics may also appear as exquisite motor behavior (Ganos et al., 2021). However, in their majority tics occur in specific body parts following a characteristic somatotopic order, and many tic behaviors are shared across different individuals (Ganos et al., 2015). Indeed, the head, the neck and shoulders are the most common body areas that exhibit tics, whereby behaviors such as blinking, eye rolling, grimacing, head jerking, or shoulder shrugging are among the most common tic behaviors. These clinical observations highlight that despite some variability in clinical presentation, tic phenomena are driven by common underlying mechanisms. This is underscored also by the fact that these same clinical characteristics are also encountered in the tic repertoires of people with other disorders, such as autism spectrum disorder (Kahl et al., 2015).

Tic phenomena can be divided into simple or complex based on the number of muscles they involve and the type of the occurring motor event. For example, single rapid motor events such as blinking, lip pulling, head tilting/jerking or shoulder shrugging are labelled simple tics. Complex tics reflect motor events that resemble goal-directed, purposeful actions, such as more complex facial expressions, hand gestures or touching objects. Prolonged tic events as part of isometric muscle contractions are referred to as tonic tics. Dystonic tics describe the occurrence of an abnormal body posture as part of a sustained tic event. In the spectrum of vocalizations, simple tics include sounds such as sniffing, throat clearing, or grunting (Mainka et al., 2019). Complex vocal tics encompass the utterance of words and (rarely) sentences, either spontaneously or as part of echo-phenomena (Ganos et al., 2012). Purposeless obscene gestures or vocalizations, also known as copro-phenomena, also belong to complex tics.

Tics have two further notable attributes. First, different from other hyperkinesias, as for example myoclonus, tics are often preceded by a sensory premonition, most commonly described as the “premonitory urge”. This means that tics do not surprise the individual who exhibits them through their occurrence. In fact, in many cases the presence of tics may often be attributed to the premonitory urge, i.e., tic behaviors may be volitionally executed in order to relieve from mounting premonitory urges (“sensory tics” (Hallett, 2002)). Although phenomenological descriptions of premonitory urges often differ between individuals (Kwak et al., 2003), each urge sensation is typically satisfied by a very specific tic behavior. Second, tics can be voluntarily suppressed (Ganos et al., 2018b). This means that effortful cognitive control may modulate the timing of tic occurrence, and on occasion (e.g., after learning as part of a behavioral treatment) also the type of tic behaviors that will emerge. On the one hand, these two features, alongside the basic phenomenological resemblance of tics to voluntary actions, have contributed to a long-lasting confusion with regard to their etiology. Indeed, psychoanalytic models to explain the presence of tic behaviors have prevailed over several decades during the 20th century and are still prevalent in certain therapeutic disciplines. On the other hand, they inform on some of the pathophysiological brain mechanisms that lead to tic emergence and their potential overlap with neural pathways involved in generation of voluntary movements.

### 2.3 The neurophysiology of tic behaviors

Neurophysiological investigations in the study of tics have had two main goals. On the one hand, they attempted to provide novel insights to tic pathophysiology. On the other hand, they sought to identify objective biomarkers associated with tic occurrence. Studies ranged from the basic characterization of muscle activation patterns of simple tic events, to measuring brain potentials from the cortical surface, and more recently also from deep nuclear brain structures.

#### 2.3.1 EMG

The neurophysiologic characteristics of a simple motor tic as recorded by surface electromyography (EMG) strongly resemble that of voluntary actions with similar burst durations (e.g., around 200 ms) and patterns of muscle activation (Hallett, 2000). For example, rapid simple motor tics exhibit the characteristic triphasic pattern of muscle activation seen in ballistic voluntary movements (Hallett, 2000). This is different from myoclonic events that may occur as single muscle bursts with shorter durations (e.g., in cortical myoclonus < 100 ms) (Shibasaki, 2006). Complex motor tics may include longer burst durations with activation patterns that may resemble that of other movement disorders. For example, the EMG pattern in dystonic tics may be indistinguishable from that recorded in people who exhibit isolated dystonia. Hence, although survey EMG may have some merit in distinguishing some tics from other brief involuntary motor events, it is unreliable as a single tool for diagnosis.

#### 2.3.2 EEG and Bereitschaftspotential

Another way to explore the neurophysiology of tic behaviors is by examining their neural antecedents. For example, routine clinical electroencephalographic investigations in people with primary tic disorders are typically unrevealing (Neufeld et al., 1990). Given the strong resemblance of tics to voluntary actions several studies attempted to examine the neural correlates that precede these two types of motor events. One established neurophysiological marker in the study of volition is the *readiness potential*, also referred to as the *Bereitschaftspotential* owing to the original electroencephalographic (EEG) studies to describe it (Kornhuber and Deecke, 1965) (Fig. 11). Although the contributing determinants to the readiness potential are a matter of ongoing debate (Schurger et al., 2021), it is generally agreed upon that it describes a ramping up of brain EEG activation a few seconds prior to the occurrence of voluntary actions with two characteristic components (Shibasaki and Hallett, 2006). The early component of the readiness potential reflects a slow increase of EEG signal negativity over both hemispheres about 1.5 s prior to the onset of a self-paced voluntary action. It is suggested to arise as a result of motor preparatory activation of the supplementary motor area and the premotor cortex (Shibasaki and Hallett, 2006). The late or lateralized component of the readiness potential is characterized by a steeper increase of signal negativity over the contralateral to the movement brain side (primary motor cortex) about 400 ms before movement onset. In primary tic disorders, three studies specifically investigated the presence and morphology of the readiness potential preceding simple motor tics (Karp et al., 1996, Obeso et al., 1981, van der Salm et al., 2012). These studies showed that not all simple tics are preceded by readiness potentials, and in the cases where readiness potentials were detected their morphology could differ. For example, van der Salm et al found the presence of readiness potentials only in simple motor tics of 6 out of 14 participants, whereas in 2 individuals simple motor tics were preceded only by the late component of the readiness potential (Karp et al., 1996, van der Salm et al., 2012). Of note, in this study self-paced voluntary actions (e.g., wrist extensions) in people with primary tic disorders were preceded, like in healthy control participants, by regular readiness potentials (van der Salm et al., 2012). Indeed, the execution of voluntary actions, as well as reaction times in basic Go/NoGo paradigms in people with TS typically fall within the normal range of motor behavior (Rae et al., 2020, Thomalla et al., 2014). These results allow to draw several important conclusions often missed in the pathophysiological discussion of tics. First, changes in readiness potentials preceding the onset of tics are specific to these motor events and do not characterize all motor behaviors of people with tic disorders. This suggests that the sources of neural signals leading to tics could differ from those of self-paced actions. Second, it appears that not all simple motor tics have the same neurophysiological correlates. This implies that distinct neurocognitive processes may underlie the manifestation of different repetitive motor behaviors captured under the tic rubric. Factors such as tic somatotopy, presence of premonitory urges and awareness over the execution of each specific tic movement could be some of the factors that drive these differences. Indeed, a range of different factors are known to influence the presence and characteristics of readiness potentials preceding voluntary actions in healthy controls (summarized in (Schurger et al., 2021)). From a clinical perspective, these studies also highlight that the utilization of the Bereitschaftspotential as a distinguishing feature of primary tics from functional movements may be unreliable, even though it can distinguish these phenomena from other jerky hyperkinesias, such as myoclonus.

A different marker used to examine the neurophysiological antecedents of tics is movement-related oscillatory brain activity. Voluntary actions are associated with changes in brain oscillations in mu- and beta-band over the sensorimotor cortex (Pfurtscheller and Lopes Da Silva, 1999). Specifically, prior and during action execution there is a characteristic decrease in the two frequencies, also known as movement- (or event-) related desynchronization, with a subsequent increase upon movement completion (movement-related synchronization). A recent EEG study looked at changes in brain oscillations in the mu- and beta-frequencies both during the spontaneous occurrence of tics and during action execution in a Go/NoGo task in adolescents and adults with TS (Morera Maiquez et al., 2022). Of note, there was no explicit mention of the type of motor tics (e.g., simple vs complex) that were captured for EEG signal analysis. This study demonstrated that different to voluntary actions in people with TS, the occurrence of tics was not associated with desynchronization changes in either of the two frequency bands. Moreover, comparison of EEG activity during the execution of voluntary actions between TS and healthy control participants also revealed group differences in the strength of movement-related signal desynchronization for the two frequency bands. On the one hand this study provides confirmatory data that the neural correlates of tics differ from voluntary actions. On the other hand, it also demonstrates that changes within the sensorimotor circuitry in people with TS extend well beyond tics and spill over to voluntary motor control. Older neurophysiological and neuroimaging studies that looked into relevant movement-related signals changes during the execution of voluntary actions in people with TS and healthy controls also corroborated this view (Biermann-Ruben et al., 2012, Franzkowiak et al., 2010, Ganos et al., 2014, Niccolai et al., 2016, Thomalla et al., 2014).

#### 2.3.3 Local field potentials

The search for tic-related neurophysiological markers and their distinguishing features from voluntary actions has been further augmented by the therapeutic application of deep brain stimulation in tic disorders, as it provided a means to record neurophysiological signals from subcortical structures. Several studies by now have demonstrated that a low-frequency oscillatory signal (between 3 and 12 Hz) recorded from both thalamic (typically from the centromedian–parafascicular nucleus CM-Pf/ in proximity to the nucleus ventralis oralis internus, Voi) and pallidal (globus pallidus pars interna) structures correlates with tic severity measures,(Bour et al., 2015, Cagle et al., 2020, Jimenez-Shahed et al., 2016, Marceglia et al., 2021, Marceglia et al., 2010, Neumann et al., 2018, Shute et al., 2016). Importantly, one study demonstrated that the duration rather than the amplitude of theta burst activity by both structures was associated with preoperative tic severity (Neumann et al., 2018). Notably, a recent investigation of both thalamic and motor cortical local field potentials in 4 subjects with TS and over a period of 6 months elegantly demonstrated the specificity of the thalamic low-frequency signal as a tic biomarker (Cagle et al., 2020). This further underscores the significance of low-frequency oscillatory brain activity in the pathophysiology of tics, and its potential toward therapeutic adaptive neuromodulation (Neumann et al., 2018).

#### 2.3.4 Transcranial magnetic stimulation

Transcranial magnetic stimulation (TMS) protocols have also been applied to probe the interaction between motor cortical pathways and tics (recently reviewed in (Latorre et al., 2019)). Specifically, TMS studies in tic disorders have provided critical insights into tic pathophysiology. First and foremost, they demonstrated an involvement of the primary motor cortex in tic expression. Indeed, several studies provided evidence for reduced excitability of the M1 in people with TS (Heise et al., 2010, Jackson et al., 2013, Orth et al., 2008), whereas clinical correlations with tic severity suggested a compensatory mechanism of the primary motor cortex to control tic output (Orth et al., 2008). In line with this were the results of a study that explored motor cortical excitability during a free ticcing and a voluntary tic inhibition state (Ganos et al., 2018a): During voluntary tic inhibition measures of motor cortical excitability, including corticospinal recruitment curves, were significantly reduced compared to free ticcing. Moreover, there was a linear relation between key neurophysiological TMS measures and the strength of tic inhibitory performance.

Driven by the pathophysiological framework that dysfunction of inhibitory interneuronal populations are a core feature of primary tic disorders (Kurvits et al., 2020), additional TMS protocols probing cortico-cortical interneuronal inhibition (e.g., short-interval intracortical inhibition: SICI), as well as inhibition of motor cortical neurons following sensory afferent pulses (i.e., short-afferent inhibition: SAI) were applied. Although reduced inhibition was demonstrated for these measures, their pathophysiological significance related to tic phenomena remains unclear. To highlight some of the complexities of result interpretation, short intracortical inhibition (SICI), for example, was characteristically reduced at baseline in people with tic disorders compared to healthy controls (Heise et al., 2010, Orth et al., 2005, Orth and Rothwell, 2009), but normalized prior to the execution of voluntary actions (Heise et al., 2010), whereas it remained unchanged during voluntary tic inhibition (Ganos et al., 2018a). Of note, both SICI and SAI have been found to be reduced in a number of hypo- and hyperkinetic movement disorders, thereby challenging their specificity to tic disorders (Latorre et al., 2019).

Motor cortical plasticity has also been the focus of several TMS studies in tic disorders as a tool to map the neurophysiology of motor learning. This was prompted both by the clinical observation that tics may be – partially – learned behaviors (Ganos et al., 2021), and the therapeutic application of behavioral interventions, such as habit-reversal training (Fründt et al., 2017). Unfortunately, the two studies that explored a specific form of associative plasticity of the M1 (also termed paired associative plasticity) in adults with TS provided contradictory results, with evidence both for and against enhanced associative plasticity of the primary motor cortex (Brandt et al., 2014, Martín‐Rodríguez et al., 2015). Some methodological differences that characterize these studies, including the clinical characteristics of participants, as well as the absence of online control over tic state (i.e., free ticcing vs volitional tic suppression) mean that it is difficult to draw firm conclusions based on these divergent results. Of note, a third study that used a different protocol to probe facilitation of the M1 following theta burst stimulation did not find evidence of enhanced neuroplasticity in TS (Suppa et al., 2011). It is important to highlight that no study has yet explored use-dependent motor plasticity, which might in fact be more ecologically valid and salient to tic occurrence and could, therefore, provide insights further insights to this aspect of tic pathophysiology.

Several other measures related to volitional and automatic behaviors further explored distinct clinical characteristics suggested to be related to specific aspects of tics. Owing to the seminal syndromic description of Gilles de la Tourette and its categorization alongside clinical entities such as Latah, Myriachit and Jumping Frenchmen under the “convulsive tic illness” spectrum (Lanska, 2017), three studies directly examined the startle reflex in people with tic disorders in the past (reviewed here (Hallett, 2002)). However, these studies did not provide a clear signal as to whether the auditory startle reflex significantly differs in people with TS (Gironell et al., 2000, Sachdev et al., 1997, Stell et al., 1995). Importantly, relevant information such as the comorbid presence of anxiety disorder in the TS participants was not provided. Of note, one further report described the clinical and neurophysiological characteristics of three cases with “late-onset startle-induced tics” (Tijssen et al., 1999). However, the specific features of these cases do not fall under the primary tic disorder rubric, but would most likely be attributed to a functional neurological disorder.

#### 2.3.5 Pre-pulse inhibition (PPI)

A different measure, related to startle response, is pre-pulse inhibition (PPI). Here, a weak sensory stimulus, the pre-pulse stimulus, precedes the presentation of a startle stimulus and leads to attenuation of the startle response (Kohl et al., 2013). In humans, PPI is typically measured through recording the EMG activation of the eyeblink component of a startle response (Kohl et al., 2013). It is suggested to be mediated through the involvement of brainstem and cortico-limbic areas and is typically used as a proxy measure of sensorimotor gating (i.e., sensory stimuli attenuate the strength of a motor response) (Kohl et al., 2013). Given that primary tic disorders are characterized not only by tics but also by the presence of several sensory symptoms, including premonitory urges and hypersensitivity to exteroceptive stimulation (Schunke et al., 2016), PPI has been the focus of several studies in both pediatric and adult populations (reviewed in (Kohl et al., 2013)). Indeed, several studies on the topic showed a reduction of PPI in TS (Castellan Baldan et al., 2014, Castellanos et al., 1996, Swerdlow et al., 2001) even though some findings were largely variable (Schleyken et al., 2020, Zebardast et al., 2013). Unfortunately, again large differences in the applied techniques and the studied populations, as well as an absence of relevant clinical correlations, do not allow making strong inferences on the pathophysiological role of PPI in TS or its application in clinics.

#### 2.3.6 Sleep

A final mention should be made to the neurophysiology of sleep in TS, as this is of interest for two specific reasons. First, the overall prevalence of sleep disturbances in TS is high with polysomnographic studies providing evidence for reduced sleep efficiency, decreased percentage of REM sleep, changes in delta sleep and increased sleep-related periodic limb movements (PLMS) (reviewed in (Jiménez-Jiménez et al., 2020)). Second, it has been reported that tics also persist during sleep (also reviewed in (Jiménez-Jiménez et al., 2020) (Jiménez-Jiménez et al., 2020)). Recent findings from an animal model of tic behaviors, namely the striatal GABAergic disinhibition model also support this observation (Vinner Harduf et al., 2021). Pharmacologically induced tic-like behaviors in rats were present both during wakefulness and sleep, even though – similar to humans - reduced during the duration of the later (Vinner Harduf et al., 2021). These results would imply that tic occurrence may be a rather automatic process, where volitional control plays only a modulatory role. However, the phenomenological and neurophysiological properties of sleep-related tics in humans, and their relation to tics during wakefulness remain understudied.

### 2.4 Discussion and summary

The study of tic behaviors, like many other neuropsychiatric syndromes, is made more difficult by the complexity and variability of clinical presentations, both with regard to the motor behaviors as well as the presence of comorbid disorders. Several of the applied neurophysiological tools discussed here provide some critical insights into the pathophysiology of tics and allow disentangling the motor phenotype from other clinical features. Specifically, they highlight the role of different input sources contributing to tic occurrence and provide some insights into the neural circuitries involved during different physiological states related both to voluntary actions and tics. In this regard, the discovery of theta-oscillatory activity in subcortical DBS targets in TS and the new venues to explore long-term cortico-subcortical interactions as performed by Cagle and colleagues (Cagle et al., 2020) further augment the pursuit for robust neurophysiological markers of tic behaviors. Such markers may be further optimized through multi-modal approaches, including connectomic-DBS, which will in turn provide a mechanistic breakdown of symptom-specific networks for therapeutic interventions (Hollunder et al., 2021). This may not only include the attenuation of one or several tic-generator networks, but also the augmentation of tic inhibitory networks, as additional tools to reduce tic severity through the application of both invasive and non-invasive neuromodulatory methods.

## 3 Startle syndromes

### 3.1 The normal startle reflex

**Fig. 13:**
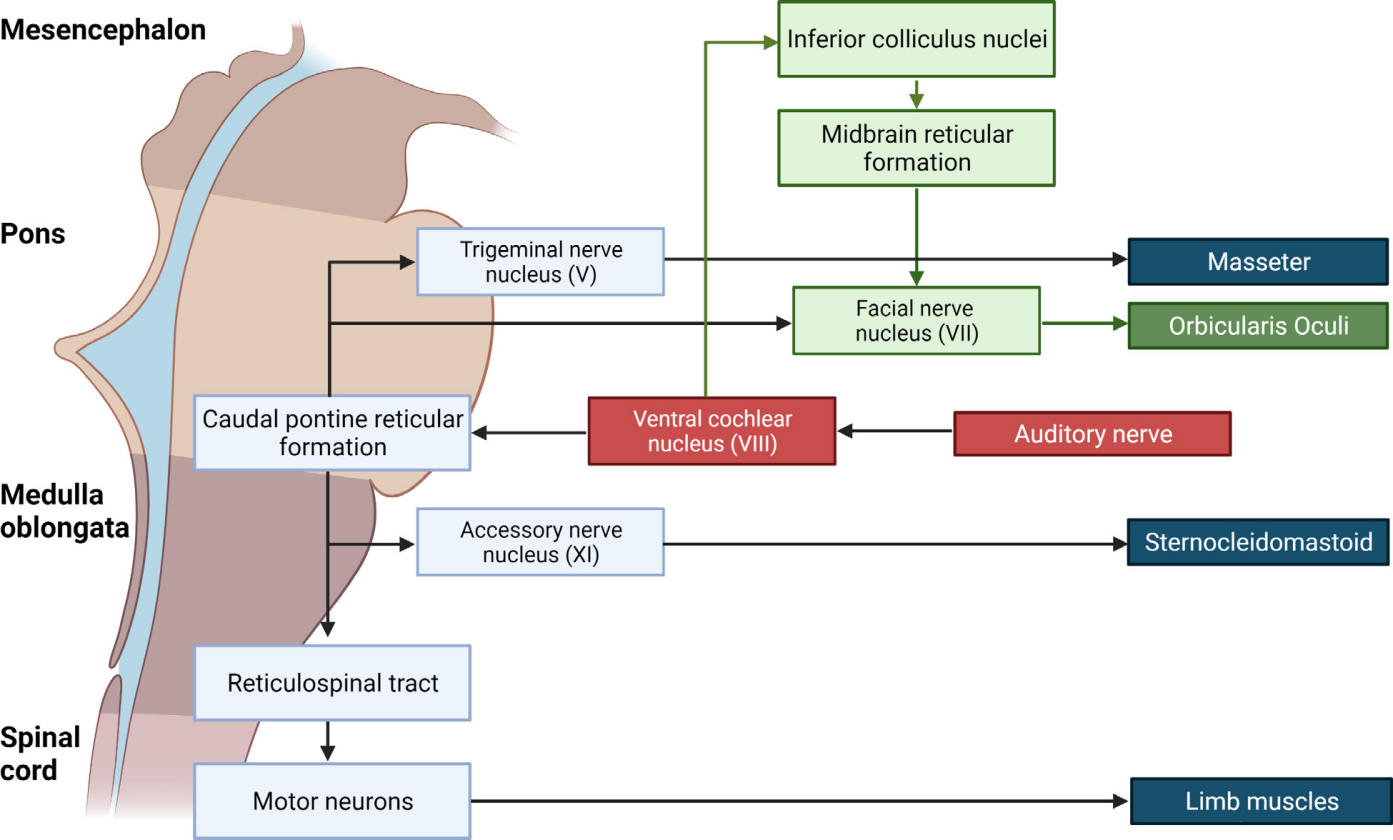
The circuit of the startle reflex and blink reflex elicit by the auditory stimulus. In red, the afferent pathway of the auditory stimulus is shown. After the signal has reached the ventral cochlear nucleus, the pathway of the blink reflex is shown in green and the startle reflex in blue. The orbicularis oculi is thought to be elicited twice: first as part of the blink reflex, second as part of the startle reflex. Created with BioRender.com. Based on (Valls-Solé et al., 2008). (For interpretation of the references to colour in this figure legend, the reader is referred to the web version of this article.)

The startle reflex is an evolutionarily old autonomous reflex, that enables an animal to respond quickly in potential threatening situations by mediating a fight or flight response and prevent injury. The startle reflex arises from the lower brainstem (Fig. 13). It is an oligosynaptic reflex originating from the medial reticular formation of the lower brainstem followed by caudal and rostral propagation causing symmetrical contraction of different muscles. The movement in a generalized startle reflex consists of eye closure, grimacing, neck and trunk flexion, slight abduction of the arms, flexion of the elbows, and pronation of the forearms (Landis and Hunt, 1939). With predominant activation of flexors, the movement that arises can be interpreted as a defensive stance with maximum postural stability. With help of electromyography, several features of the normal startle reflex have been described including latency of onset, duration, magnitude, muscle recruitment, and habituation. The reflex and effects of its modulation have been extensively studied with help of eliciting the reflex by an auditory stimulus (Dreissen and Tijssen, 2012). The normal auditory startle reflex consists of two responses, a fairly consistent early response or motor reflex and a more variable second response (Brown et al., 1991b).

The auditory startle reflex and its associated studies, especially pre-pulse inhibition (PPI; see Section 2.3.5), have been used in fundamental research focused on the sensorimotor gating mechanisms of the central nervous system in various neurological and psychiatric disorders, including parkinsonian syndromes, dystonia, tics disorders, functional movement disorders, schizophrenia, and obsessive–compulsive disorders (Gironell et al., 2000, Gómez-Nieto et al., 2020, Kiziltan et al., 2015, Schleyken et al., 2020, Valldeoriola et al., 1998, Vidailhet et al., 1992). In some disorders (e.g., post-traumatic stress disorder, minimally conscious state), it has found a place amidst the diagnostic process (Glover et al., 2011, Hermann et al., 2020). Most of these patients do not exhibit a clinical exaggerated or excessive startle reflex and are therefore not considered startle syndromes. However, electromyographic features measuring habituation, magnitude, spread, and velocity can be abnormal compared to healthy controls.

#### 3.1.1 The early response

**Fig. 14:**
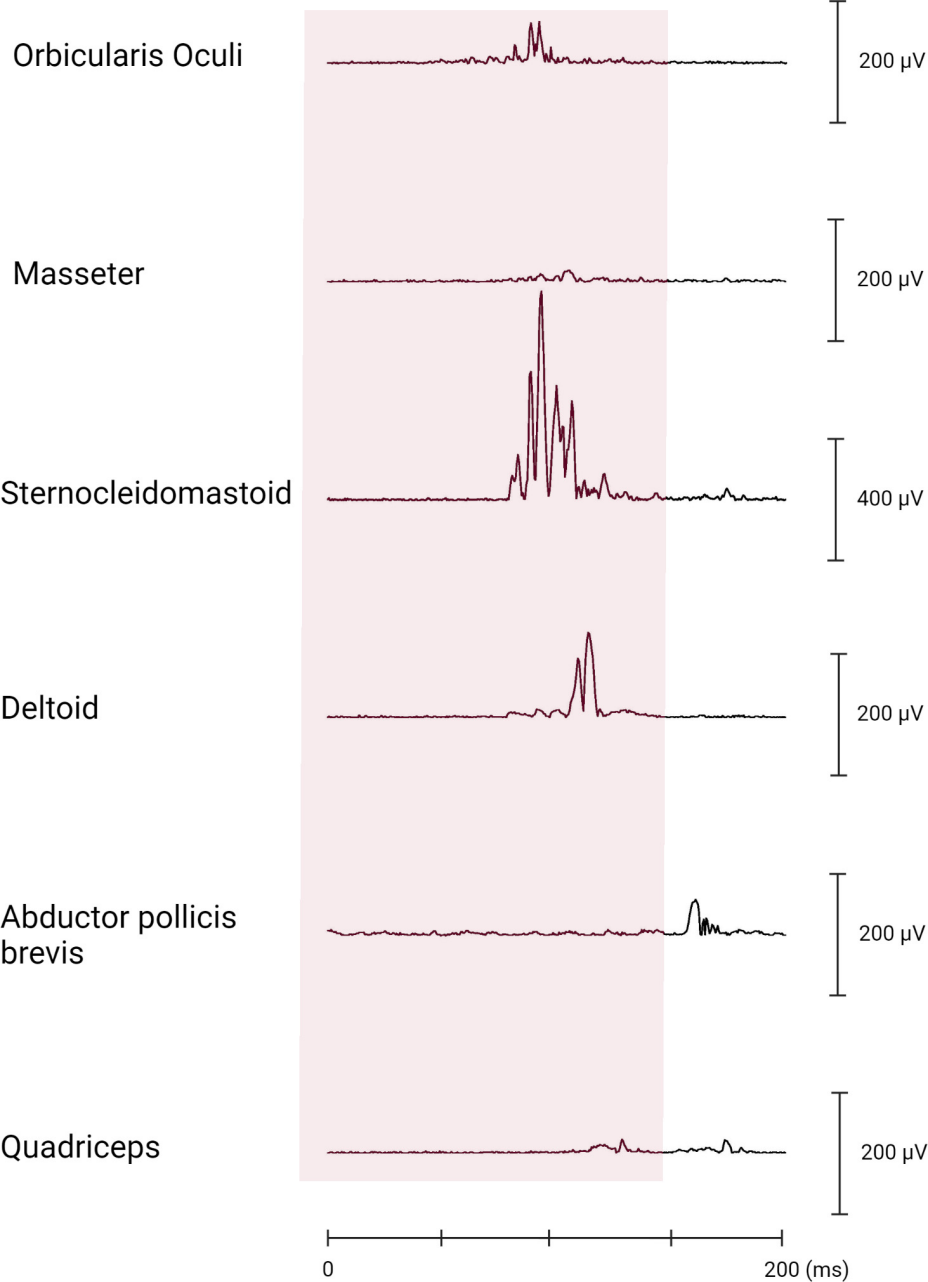
Rectified electromyographic (EMG) (single trial) muscle responses of a 13-year-old boy following 104 dB tone. The latency of the abductor pollicis brevis (APB) muscle is disproportionately long as the onset is seen after the quadriceps response Although not explained in the original article, the auditory blink reflex does not seem to be present in this EMG (Bakker et al., 2009; Dreissen et al., 2012).

The early response consists of initial activation of the sternocleidomastoid with a *latency to onset* of ± 50 ms (58.3 ms, range 40.4–136 ms; n = 53 in Brown et al., 1991a, Brown et al., 1991b, Brown et al., 1991c, Brown et al., 1991d), followed by rostral propagation towards the seventh then fifth cranial nerve nuclei causing contraction of the mentalis and orbicular oculi then the masseter (Fig. 14). The caudal propagation causes distance-dependent segmental activation of trunk and limb muscles, with synchronous activation of antagonist muscles ending within ± 400 ms after onset (Fig. 14) (Brown et al., 1991b, Dreissen and Tijssen, 2012). One exception within the latencies of onset are those of the intrinsic hand muscles as they are disproportionately delayed, 25 ms after the forearm extensors, due to the fact that the reticulospinal tract, and not the corticospinal tract mediates the response (Brown et al., 1991b). Although the rostro-caudal pattern of *muscle recruitment* is similar between individuals, the extent varies and, in one study, is limited to the sternocleidomastoid in a third of people. Only in a small proportion of people fully generalized activation with involvement of the leg muscles occurs (Brown et al., 1991b). The extent of the muscle recruitment can be represented by the muscle response probability, being the chance that a particular muscle responds following stimulus (Bakker et al., 2006). *Habituation* of the startle reflex occurs fast, especially compared to the auditory blink reflex, within 2–5 trials and is associated with shortening of the reflex burst duration (Brown et al., 1991b, Wilkins et al., 1986). Habituation can be measured by visual inspection or objectively by decrease of the area under the rectified EMG, representing duration and amplitude, over consecutive stimuli (Chokroverty et al., 1992).

The auditory blink reflex of the orbicularis oculi is not part of early phase of the auditory startle reflex, but precedes it (Brown et al., 1991b). The auditory blink reflex is a brief muscle contraction of the orbicularis oculi in response to sound stimuli. Originating from the midbrain reticular formation (Fig. 13), the *latency to onset* of the blink reflex is about 30–40 ms after auditory stimulus with a second component in the EMG response coinciding with the muscle recruitment of the auditory startle reflex (Brown et al., 1991b, Wilkins et al., 1986). Compared to the startle reflex, the duration of the blink reflex being about 60–150 ms is brief and shows far less habituation (Brown et al., 1991b, Landis and Hunt, 1939).

#### 3.1.2 The second response

**Fig. 15:**
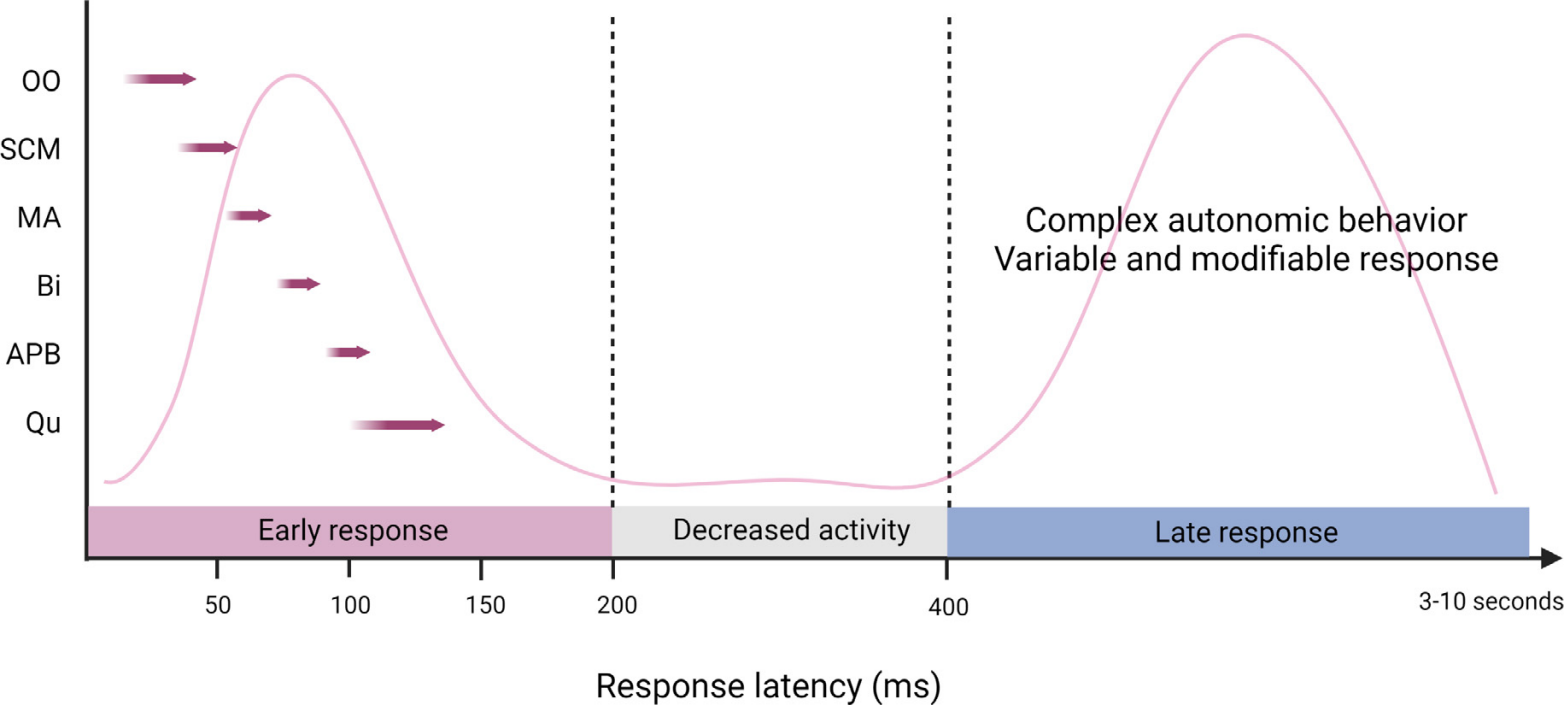
A schematic diagram to depict the early and late components of the typical normal human startle response. The sequence of muscle recruitment consisting of the orbicularis oculi (OO), the sternocleidomastoid (SCM), masseter (MA), biceps (Bi), abductor pollicis brevis (APB) and quadriceps (Qu) can be seen with a latency to onset between 20 and 150 ms. No electromyographic (EMG) activity is visible in the period between the early and late response (200–400 ms). No typical muscle recruitment exists for the late response lasting up to 3–10 s, as this is thought to be complex autonomic behavior. Created with BioRender.com. Based on (Saini and Pandey, 2020).

The second response or orienting response has less well-defined electrophysiological markers, as this shows more variability between individuals and is considered to be a complex automatic behavior to prepare for defense or attack (Davis, 1948, Valls-Solé et al., 2008, Wilkins et al., 1986). It is stated by Pavlov as the ‘What is it?’ reflex (Pavlov, 1927). After a *latency* period of 250–300 ms with decreased activity that follows the early response, the second response can be observed with a *burst duration* of 3–10 s or more. It is influenced by the psychological state resulting in postural adjustments and orientation towards the stimulus (Fig. 15) (Gogan, 1970). Although a fair correlation exist between the *amplitude* of the early and orientation response, both can be independently present (Gogan, 1970). In contrast to the early response, no pattern is seen in the *muscle recruitment* but rather complex and behavioral activation. *Habituation* occurs faster than the early response (Gogan, 1970).

#### 3.1.3 The autonomic response

During and after the second response, autonomic responses can be measured including an increased galvanic skin response (i.e., electrodermal changes of sweat gland activity), increased systolic blood pressure, pupillary dilatation, and heart rate (Dreissen and Tijssen, 2012).

#### 3.1.4 Elicitor of the startle

The startle reflex can be elicited by visual, somatic and tactile stimuli, but most commonly an auditory stimulus has been used (Brown, 1995, Dreissen et al., 2012). A strike of wood upon wood or a pistol shot was used in early investigations to generate an unexpected auditory stimulus, until the first electrophysiological ‘startle’ protocol was introduced in 1986 to enable elegant clinical observations. It consists of a 1000 Hz tone burst of 10 ms duration at an intensity of 108 dB, randomly once every-five minutes (Wilkins et al., 1986). Six years later an updated technique was presented, including a binaural 105 dB tones delivered in 5 blocks of 4 tones. Successive blocks were separated by a 5 min period without tones stimuli and had progressively shorter inter-stimulus intervals (ISIs) beginning with 5 min between stimuli in the first block and reducing to 1 min between stimuli in the final, fifth block (Chokroverty et al., 1992). More recent studies have elicited the startle reflex with a standardized binaurally auditory tone burst of 1000 Hz frequency of 50 ms at 124 dB through earphones. This stimulus is given randomly once every 20 min while relaxed (Brown et al., 1991b).

#### 3.1.5 Modifying influences

Multiple modulators of the startle reflex have been investigated, although the pathways of many of these modifying influences are still unknown (Dreissen et al., 2012). The magnitude or amplitude of the startle reflex can be influenced by 1) the stimulus, 2) conditioning of the subject, 3) individual characteristics of the subject, 4) and medication.

First, variability in the magnitude of the early response is in proportion with the strength of acoustic stimulus (Davis, 1948, Gogan, 1970). Second, preconditioning by means of a weak pre-stimulus reduces the reflex magnitude (Silverstein et al., 1980). The magnitude is influenced by the uncertainty or unexpectedness of the stimuli, the position of a subject, and loading (Dreissen and Tijssen, 2012, Heckman and Perreault, 2019). The position of the subject can influence the magnitude of the response by letting the subject perform a pre-existing voluntary isocontraction, which will induce a superimposed early response followed by EMG silence period of 100–130 ms (Davis, 1948, Rossignol, 1975). The influence of loading has been studied with help of the subject’s body weight, the startle reflex occurs more frequently during standing compared to sitting or standing with body weight support (i.e., bilateral unloading) (Nonnekes et al., 2013). Third, individual features that have been suggested to influence the startle reflex are changes in an individual valence (e.g., with imagery, emotional associations, or odor), attentional focus, wakefulness (e.g., a decreased reflex response in NREM state), and auditory disorders (Bohlin and Graham, 1977, Ehrlichman et al., 1995, De Groote et al., 2020, Lang et al., 1990, Silverstein et al., 1980). An increased auditory startle response has been associated with reduced sound tolerance, hyperacusis, and tinnitus (De Groote et al., 2020). Fourth, pharmacological tuning has been tried to enhance the startle reflex with local 5-OH-tryptophan, noradrenaline, and dopamine, or to decrease the response with cyproheptadine, and clonidine, without effect of acetylcholine (Wilkins et al., 1986).

### 3.2 Phenomenologically similar physiological reflexes

Other physiological reflexes originating in the caudal brainstem reticular formation can phenomenologically resemble a startle reflex and include a spino-bulbo-spinal reflex, and a trigeminocervical reflex (Merchant et al., 2019). The spino-bulbo-spinal reflex occurs after cutaneous nerve stimulation, has mostly been studied in animals and shows a total body jerk-like movement of predominantly flexors (Shimamura et al., 1964). No clear associations between neurological disorders and an exaggerated spino-bulbo-spinal reflex have been described. The trigeminocervical reflex is elicit by noxious stimulation in the trigeminal innervation area resulting in neck withdrawal due to contraction of the extensor *m. splenius* (Ertekin et al., 2001). Its pathophysiological variant, an exaggerated trigeminocervical reflex with lack of habituation, is thought to represent the head retraction reflex and can be provoked by gentle taps to the glabella, nose ridge, upper lip, and chin. Apart from being present in startle syndromes (see Section 3.3), it has been seen in patients with Niemann-Pick type C and Parkinson’s disease, although the latter remains unconfirmed (Martin et al., 2020, Sandyk et al., 1982). The clinical resemblance lies in the characteristic rostro-caudal propagation with the relative slowly conducting efferent pathways. Differences can be seen in the type of afferent stimulus inducing the reflex, the dominant involved muscles (flexors versus extensors), and associated neurological disorders when exaggerated.

### 3.3 Startle syndromes

The startle syndromes have been clinically subdivided in three groups including the exaggerated startle reflex (e.g., hyperekplexia, stiff person syndrome), the neuropsychiatric disorders (e.g., functional and cultural startle syndromes), and the stimulus-induced disorders (e.g., reflex myoclonus) (Bakker et al., 2006, Dreissen and Tijssen, 2012).

#### 3.3.1 The exaggerated startle reflex

The startle reflex results in the early response in eye closure, grimacing, neck and trunk flexion, slight abduction of the arms, flexion of the elbows, and pronation of the forearms. An exaggerated startle reflex shows the same patterned response but is poorly habituating. Furthermore, excessiveness of the startle can be seen in different electromyographic features, including an excessive burst duration and amplitude, prolonged onset latencies or a more widespread muscle activation (Dreissen et al., 2012, Wilkins et al., 1986). An exaggerated startle reflex can have a genetic cause or can be acquired. The genetic form is known as hereditary hyperekplexia, the acquired forms consist of auto-immune disorders in the stiff-person spectrum. Furthermore, exaggerated startle reflexes can occur secondary to lesions of the cerebrum or brainstem. These lesions can also be caused by genetic disorders.

##### 3.3.1.1 Hyperekplexia

Phenotype and genotype

Hereditary hyperekplexia is a genetic disorder characterized by a clinical triad consisting of 1) generalized stiffness immediately after birth, normalizing during the first year of life; 2) an abnormal startle reflex to unexpected, particularly auditory, stimuli that is present from birth; and 3) a short period of generalized stiffness following the startle response, during which voluntary movements are impossible (Tijssen et al., 1995). An exaggerated head retraction reflex, periodic limb movements during sleep (PLMS) and hypnagogic myoclonus have been described as associated features (Dreissen et al., 2012). The vast majority of patients (61–63 %) show a molecular defect in the GLRA1 gene, with the remaining in the GLRB (12–14 %) and SLC6A5 (25 %) gene, causing dysfunction of glycinergic inhibitory transmission (Saini and Pandey, 2020, Tijssen and Rees, 2012).

**Exaggerated startle in hyperekplexia**

The pattern in the *muscle recruitment* and slow efferent conduction velocity (69 m/s, range 34–133 m/s) is similar between healthy controls and hyperekplexia patients, representing a true startle reflex (Matsumoto et al., 1992, Oguro et al., 2005, Tijssen et al., 1997). The differences of the exaggerated startle reflex in hyperekplexia compared to the normal startle reflex include an increased *frequency* of occurrence, occurrence of the startle reflex at less intense acoustic stimuli, increased *amplitude*, and a tendency to shorter *latencies of onset*. An increased objective *habituation* is seen over trials, which is defined a larger reduction in amplitude and duration compared to healthy controls. However, the subjective habituation of the startle reflex over trials is impaired as the initial reflex is high in amplitude and the level of amplitude in the healthy controls is not reached within the stimulus period of the experiments (Brown et al., 1991c, Matsumoto et al., 1992, Tijssen et al., 1997, Wilkins et al., 1986). There are no EMG studies assessing the features of the generalized stiffness during the orienting phase; however, an increased galvanic skin response during the autonomic response has been reported (Tijssen et al., 1997).

Apart from the exaggerated auditory startle reflex, the head retraction reflex after tactile stimulation can be seen during neurological examination as well as with neurophysiological testing, showing abnormal short onset latencies (<25 ms) in the *m. trapezius* after electrical stimulation of the trigeminal nerve (Brown et al., 1991c, Khasani et al., 2004, Matsumoto et al., 1992, Oguro et al., 2005, Saini et al., 2018). By tactile stimulation of the median nerve to investigate the long latency reflex of the abductor pollicis, the presence of the C-reflex (e.g., a delayed response) can be noticed (Markand, 1984). Normal somatosensory and brainstem evoked potentials were recorded (Matsumoto et al., 1992). No spike or abnormal cerebral activity is present with EEG (Markand, 1984, Matsumoto et al., 1992).

**Minor form**

A minor form of the genetic disorder hyperekplexia was thought to exist, characterized by only an excessive startle reflex without stiffness. No habituation, by means of reduction in response size, with repetitive stimuli was seen in these individuals and no genetic variants were found. Neurophysiological testing in this group revealed prolonged latencies in the orbicularis oculi, the sternocleidomastoid, and biceps, suggesting a behavioral origin, and not a true startle reflex (Tijssen et al., 1996). Furthermore, normal reciprocal inhibition of the H-reflexes after electrical stimulation were found. This indicates normal spinal motor excitability consistent with lack of stiffness (Dreissen and Tijssen, 2012). Based on these findings, the minor form of hyperekplexia is thought to represent a learned startle reflex resulting from observing family members with hyperekplexia (Bakker et al., 2006).

##### 3.3.1.2 The stiff-person spectrum disorders

**Phenotype**

An exaggerated startle reflex can be seen in acquired auto-immune disorders, especially the stiff-person spectrum disorders. Stiff-person syndrome is characterized by fluctuating stiffness in the legs and axial lumbar area, stimulus-sensitive spasms, abnormal axial posture, and an exaggerated startle reaction (Brown and Marsden, 1999). Other syndromes that are part of the stiff-person spectrum disorders include the variants stiff-person plus, the aforementioned signs combined with additional neurological symptoms, and progressive encephalitis with rigidity and myoclonus, with a potentially fatal clinical course (Balint et al., 2018). Compared to the transient stiffness following a startle reflex in hyperekplexia, the stiffness in stiff-person syndrome is nearly continuous (Dreissen et al., 2012). The autoantibodies that have been linked to this spectrum are directed against glutamic acid decarboxylase, the glycine receptor type 1 and 2, amphiphysin, dipeptidyl peptidase-like protein 6, and γ-aminobutyric acid type A receptor (Balint et al., 2018). Especially autoantibodies against the glycine receptor (GlyR), disrupting the functionality of the same glycine-gated chloride channels involved in hyperekplexia, present with an exaggerated startle reflex as it is seen in 57 % of GlyR-positive patients (Carvajal-González et al., 2014, Rauschenberger et al., 2020).

**Exaggerated startle in stiff-person syndrome**

Exaggerated startle reflexes in patients with stiff-person syndrome show the *latency* of onset and pattern of *muscle recruitment* similar to the normal startle reflex but with poor *habituation* and excessive motor activity, especially in the axial and leg muscles (Matsumoto et al., 1994). The exaggerated head-retraction reflex is present (Khasani et al., 2004).

##### 3.3.1.3 Other clinical syndromes

Other patients presenting with an exaggerated early response of the startle reflex are often caused by acquired damage to the cerebrum or brainstem (Bakker et al., 2006). Furthermore, a few genetic disorders other than hyperekplexia have been described to present with an exaggerated startle reflex as part of a more elaborate clinical syndrome. An overview can be found in a recent review by Saini and Pandey (Saini and Pandey, 2020). Unfortunately, the majority of case reports do not state electrophysiological data of the presumed exaggerated startle reflex and did not study habituation. Furthermore, a number of these case reports have referred to the presence of an exaggerated startle reflex as hyperekplexia; however, the term hyperekplexia should be reserved for the distinct clinical syndrome (see Section 3.2.1.1) caused by a molecular defect in the glycine-ligated chlorine channel (Brown et al., 1991c, Dreissen and Tijssen, 2012, van der Veen et al., 2020).

#### 3.3.2 Neuropsychiatric startle disorders

Neuropsychiatric startle disorders are thought to show an exaggerated second response of the startle reflex, the orienting response. However, the orienting response is more difficult to study systematically due to its variability and behavioral influences. Nevertheless, functional startle syndromes and various culture-specific syndromes are considered to present predominantly with an exaggerated orienting response.

##### 3.3.2.1 Functional startle syndromes

Functional startle syndromes are part of the large heterogeneous category of functional movement disorders (Edwards and Bhatia, 2012). In a study on the startle reflex in functional movement disorder patients, the early response of the startle reflex showed an enlargement of the *amplitude* probably due to hyperarousal). However, most discriminatory, an enlarged *response probability* was seen in both the early motor and the orienting response. In seventeen patients with a functional movement disorder, the total response probability was 17.1 % (*SD* ± 18.6) in the early and 21.6 % (*SD* ± 17.9) in the orienting response compared to 8.9 % (*SD* ± 7.5) and 5.5 % (*SD* ± 6.1) in healthy controls. Interestingly, a patient with functional abdominal jerks showed a higher response rate of the rectus abdominis muscle compared to healthy controls, which was hypothesized to represent a start-react phenomenon (Dreissen et al., 2017). This phenomenon is the release of a prepared and stored movement from the cortex at faster latencies due to an involuntary subcortical trigger, in this case the auditory startle reflex (Stevenson et al., 2014). Dreissen and colleagues hypothesized that the patients with functional abdominal jerks are constantly subconsciously ‘preparing’ (demonstrated with the presence of a Bereitschaftspotential) of their upcoming jerk, which are released by a startling stimulus.

##### 3.3.2.2 Various culture-specific syndromes

There are various culture-specific syndromes which can be described as non-habituating hyper startling of the second – the behavioral – response following the normal habituating early motor startle response. Peculiar behaviors have been observed evoked by loud noises or being poked forcefully in the side in different locations around the world (Simons, 1996). Each of these geographic-specific responses seem to be colored strongly by culture, have been given a local name and have been embedded in local traditions. Examples are the Jumping Frenchmen in North America among French-Canadian descendants (Richard, 2018), the Latah in Southern Asia (Bakker et al., 2013), and the Miryachit in Siberia (Hammond, 1884). A case series of the Latah patients described the overlap and difference with tic disorders (e.g., the presence of sensitization, echo phenomena, palilalia, and coprolalia; but absence of spontaneous movements and sensation of relief in majority) and functional movement disorders and concluded this entity to be a neuropsychiatric startle syndrome. The functional origin is supported by features including the paroxysmal nature, sudden onset, suggestibility of the patients, and frequent presence of a stressful dream or other event previous to the onset of the bizarre startle movements (Bakker et al., 2013). Notwithstanding anthropological, psychodynamic and neurobiological research, the categorization of all cultural-specific startle syndromes is still an ongoing debate being either a behavioral phenomenon belonging in the cultural or anthropological realm, or a neurological disorder of which the expression is prone to local cultural influences (Dreissen et al., 2012, Lanska, 2017).

#### 3.3.3 Startle-induced disorders

Sudden stimuli can evoke excessive responses other than startle but phenomenologically closely resembling an excessive startle reflex. These startle-triggered syndromes are classified as stimulus or startle-induced disorders. These include mainly reticular reflex myoclonus and cortical reflex myoclonus. Other startle-induced disorders can be epilepsy, narcolepsy, paroxysmal kinesiogenic dyskinesia, and tics (Dreissen et al., 2012, Tijssen et al., 1999).

##### 3.3.3.1 Startle epilepsy

Startle epilepsy is a rare form of epilepsy comprised of epileptic seizures provoked by unexpected stimuli most often seen pre- or perinatal in patients with a variety of localized or diffuse static brain pathology (Panayiotopoulos, 2005). The epileptic startle responses occur multiple times per day, last up to 30 s and cause falls due to axial tonic posturing. In a minority of patients, the seizures are asymmetrical, atonic or myoclonic. Autonomic manifestations, automatisms, laughter and jerks may co-occur. Spontaneous seizures are common in these patients but are infrequent compared to the startle seizures. Interictal EEG abnormalities related to the underlying epileptic disorders can be present in startle epilepsy. Ictal EEG can help differentiate between an exaggerated startle reflex showing no abnormalities compared to a startle seizure with an initial vertex discharge followed by diffuse relative flattening or low voltage rhythmic approximately 10 Hz activity spreading to the mesial frontal, parietal and contralateral frontal region (Manford et al., 1996).

### 3.4 Discussion and summary

In summary, the startle reflex is an old defensive strategy originating from the brainstem optimizing the fight or flight response of animals. In healthy subjects the startle reflex can be exaggerated due to modulating by factors, such as conditioning or emotional state, causing excessive startles. A pathological persistence of either the early or late response of the reflex is associated with several neurological disorders. An exaggerated early response and impaired habituation is seen in the hereditary startle syndrome hyperekplexia and several acquired startle syndromes such as the stiff-person auto-immune disorder. The term hyperekplexia should be reserved for the distinct clinical syndrome characterized by the exaggerated startle reflex followed by a short period of generalized stiffness, in combination with the presence of generalized stiffness in the first year of life. It is important to distinguish the disorders with exaggerated startle reflexes from neuropsychiatric startle and startle-induced syndromes for which clinical neurophysiology is a useful diagnostic tool. Electromyographic studies can help differentiate between these startle syndromes focusing on the presence of habituation, amplitude of the muscle activity, muscle recruitment, and latencies of onset after the elicitor.

## 4 Restless legs syndrome and periodic leg movements during sleep

### 4.1 Introduction

Restless legs syndrome (RLS) is a common sensorimotor neurologic disorder, affecting up to 10 % of the general population in Europe and North America (Berger et al., 2004, Hogl et al., 2005, Ulfberg et al., 2001a, Ulfberg et al., 2001b). RLS represents a significant personal and social burden, at least in Europe and the US (Durgin et al., 2015, Trenkwalder et al., 2021).

Periodic leg movements during sleep (PLMS) are present in up to 80 % of patients with RLS (Montplaisir et al., 1997), but can be found also in healthy subjects (Pennestri et al., 2006). Rarely, PLMS in the absence of RLS or other sleep disorders might cause a clinically significant sleep disturbance or impairment in important areas of functioning, a condition called periodic limb movement disorder (PLMD) (American Acedamy of Sleep Medicine, 2014).

In this section, we will address RLS and PLMS. We will provide definitions, diagnostic criteria, and epidemiological data, and present current knowledge about pathophysiological mechanisms. Moreover, we will specifically focus on assessment methods and neurophysiological correlates.

### 4.2 Restless legs syndrome

#### 4.2.1 Diagnosis

Restless legs syndrome (RLS) is a sensorimotor disorder often described as an unpleasant sensation during rest, associated with an urge to move the legs and sometimes the arms, which begins or worsen in the evening or at night and improves with movement (Allen et al., 2014). The diagnosis of RsLS is made through clinical evaluation and exclusion of mimics.

In 1995, the International Restless Legs Syndrome Study Group (IRLSSG) developed the first diagnostic criteria based on a broad international consensus of clinical RLS experts (Walters et al., 1995). These underwent a first revision in 2003, when the wording was changed to improve clarity (e.g., “motor restlessness’’ was replaced by ‘‘urge to move,’’), the relief of symptoms by movement became a separate criterion and specific diagnostic criteria were introduced for children and cognitively impaired elderly (Allen et al., 2003). In 2014 further changes were made to improve specificity, with the addition of a fifth criterion to exclude possible mimic conditions (Allen et al., 2014). In addition, specifiers for the clinical course and clinical significance were introduced. Regarding clinical course, a distinction between chronic-persistent RLS (symptoms when not treated would occur on average at least twice weekly for the past year) and intermittent RLS (symptoms when not treated would occur on average < 2/week for the past year, with at least five lifetime events) has been made. For what concerns clinical significance, this had been defined when RLS causes significant distress or impairment in social, occupational, educational, or other important areas of functioning by their impact on sleep, energy/vitality, daily activities, behaviour, cognition, or mood. The current IRLSSG diagnostic criteria for RLS are listed in Table 2 (Allen et al., 2014).

**Table 2:** The current International Restless Legs Syndrome Study Group (IRLSSG) diagnostic criteria for restless legs syndrome (Allen et al., 2014).

An urge to move the legs usually but not always accompanied by or felt to be caused by uncomfortable and unpleasant sensations in the legs
The urge to move the legs and any accompanying unpleasant sensations begin or worsen during periods of rest or inactivity such as lying down or sitting.

The urge to move the legs and any accompanying unpleasant sensations are partially or totally relieved by movement, such as walking or stretching, at least as long as the activity continues.

The urge to move the legs and any accompanying unpleasant sensations during rest or inactivity only occur or are worse in the evening or night than during the day.

The occurrence of the above features is not solely accounted for as symptoms primary to another medical or behavioral condition (e.g., myalgia, venous stasis, leg edema, arthritis, leg cramps, positional discomfort, habitual foot tapping).

#### 4.2.2 Epidemiology

Overall prevalence of RLS has been estimated to be 5–10 % in European and North American populations (Berger et al., 2004, Didriksen et al., 2017, Hogl et al., 2005, Ulfberg et al., 2001b, Ulfberg et al., 2001a). In these countries, around 3 % of RLS patients have clinically significant (i.e., symptoms at least twice a week, at least moderate distress) RLS (Allen et al., 2010, Allen et al., 2005). In Asian countries prevalence is reported to be below 2 % (Cho et al., 2009, Nomura et al., 2008, Sunil et al., 2007). Epidemiological data on RLS in other populations are sparse.

In women prevalence of RLS is about twice as high than in men (American Acedamy of Sleep Medicine, 2014). Prevalence increases with age, up to the age of 60–70 years (American Acedamy of Sleep Medicine, 2014).

#### 4.2.3 Pathogenesis

The pathophysiology of RLS is complex and has been linked to altered dopaminergic neuro-transmission and brain iron deficiency in the context of a heterogeneous genetic background (Allen, 2015, Trenkwalder et al., 2018). Dopaminergic dysfunction has been suggested in RLS, with a “hyperdopaminergic” presynaptic state and a “hypodopaminergic” postsynaptic state. Another mechanism involved in the pathogenesis of RLS is a CNS iron deficiency, with consequent inadequate iron transport across the blood–brain barrier and subsequently a failure to import adequate iron into critical neuronal cells (Allen, 2015). Most of the early-onset cases of RLS present a positive family history. A meta-analysis of genome-wide association studies confirmed *MEIS1* (which plays an important role in the development of the central and peripheral nervous systems) to be the strongest genetic risk factor for RLS (Schormair et al., 2017). Additionally, a multimodal load analysis detected RLS associations of 14 genes, mostly with functions in calcium transport and neurogenesis (Tilch et al., 2020). In elderly people, RLS is often associated with comorbidities such as cardiovascular disease, obstructive sleep apnea syndrome, diabetes, or neuropathy. Therefore, RLS can be seen as a multifactorial disease with a continuous spectrum, where genetic contribution plays a major role in early onset cases and environmental factors or comorbid diseases contribute increasingly to the pathogenesis with advancing age (Trenkwalder et al., 2018, Trenkwalder et al., 2016).

Peripheral hypoxia has also been reported to be a contributing etiologic factor in RLS pathogenesis (Castillo et al., 2006, Gupta et al., 2017, Koh et al., 2015, Larsson et al., 2007, Patton et al., 2011, Salminen et al., 2014, Wahlin-Larsson et al., 2009).

#### 4.2.4 Neurophysiological correlates

##### 4.2.4.1 Periodic leg movements in RLS

In up to 80 % of patients with RLS periodic leg movements (PLM) are present (Montplaisir et al., 1997). Specific criteria have been established to detect PLM (Berry et al., 2020, Ferri et al., 2016). These will be addressed in the next section, specifically dedicated to PLM.

PLM during sleep (PLMS) are defined as a PLMS index of more than 15/h. In patients with RLS, PLMS present a night-to-night variability and are more frequent during the first half of the night. PLMS are often related to arousals, thus contributing to sleep disturbances in RLS patients (American Acedamy of Sleep Medicine, 2014). The clinical relevance of PLMS will be further addressed in the next section.

##### 4.2.4.2 Painful sensations and small fiber neuropathy

Patients with RLS present by definition unpleasant leg sensations, and a subgroup of patients even refer pain (Karroum et al., 2015). The mechanisms underlying these abnormal leg sensations are still incompletely understood. A spinal dopamine dysfunction has been suggested, with central sensitization and dysfunction of descending dopaminergic diencephalo-spinal pathways involved in the spinal control of nociception, leading to mechanical hyperalgesia (Clemens et al., 2006). However, more recent studies suggest that RLS is a network disorder, with a widespread involvement of the central and peripheral nervous system (Lanza et al., 2017). In line with this concept, recent brain imaging studies on a large cohort of RLS patients reported with matter reduction in key somatosensory circuits and structural alterations of the frontopontine tract (Stefani et al., 2019), as well as alterations in functional connectivity among regions associated with processing of sensory information (Tuovinen et al., 2021).

At least in some patients, the presence of subclinical sensory neuropathy has been proposed as RLS trigger, through abnormal sensory inputs and sensitization of the dorsal horns (Bachmann et al., 2010, Gemignani et al., 2013, Gemignani et al., 2009, Lim et al., 2012, Polydefkis et al., 2000). These are usually late-onset cases without a family history of RLS (Polydefkis et al., 2000). In patients with RLS associated with small fiber neuropathy thermal hypoesthesia to cold and warm, as well as hyperalgesia to pinprick have been reported (Bachmann et al., 2010). A recent study underlines that skin biopsy should be performed to assess the presence of small fiber neuropathy, according to current diagnostic criteria (Bergmann et al., 2021). Using this methodology, the coexistence of RLS and small fiber neuropathy seems to be rare. Of note, skin biopsy showed increased expression of tyrosine hydroxylase in dermal nerve bundles in patients with RLS, suggesting enhanced peripheral adrenergic activity (Bergmann et al., 2021). These findings are in line with the proposed concepts of postganglionic sympathetic disinhibition in RLS (Walters and Rye, 2009).

Although the pathophysiological mechanism causing painful sensations in RLS and the causal relationship with peripheral nerve damage are still unclear, a diagnosis of comorbid neuropathy is fundamental in patients with RLS, as it allows an optimized treatment efficacious on both conditions.

#### 4.2.5 Assessment of RLS symptoms

##### 4.2.5.1 Severity scales

To assess the severity of RLS symptoms, rating scales have been developed and validated. The most used are the International RLS Study Group rating scale (Walters et al., 2003) (IRLS, also validated as self-administered version sIRLS) (Sharon et al., 2019), the Johns Hopkins RLS severity scale (JHRLSS) (Allen and Earley, 2001), and the RLS-6 scale (Kohnen et al., 2016). A specific scale for assessment of symptom severity in children has also been developed (Arbuckle et al., 2010, Walters et al., 2014).

##### 4.2.5.2 Suggested immobilization test

The suggested immobilization test (SIT) is a standardized test originally developed with the aim to provide an objective method to diagnose RLS (Montplaisir et al., 1998). This test evaluates PLM and self-reported sensory symptoms for people who are instructed to remain still for one hour, while sitting on a bed with their legs outstretched. Quantification is performed by recording the anterior tibialis muscles with surface EMG, counting all movements lasting 0.5–10 s, separated by an interval between 4 and 90 s and occurring in a series of four consecutive movements. The SIT PLM index represent the number of periodic leg movements per hour of immobility. The visual analog scale was obtained every-five minutes to measure the perceived leg discomfort (Michaud et al., 2002).

However, the SIT presents some intrinsic limitations due to the single administration time, being not able to evaluate any symptoms occurring outside its administration time. To overcome this limitation, the multiple SIT (mSIT) has been validated and can be useful in evaluating RLS severity and treatment response (Garcia-Borreguero et al., 2013).

### 4.3 Periodic leg movements

#### 4.3.1 Definition

Periodic leg movements (PLM) are periodic episodes of repetitive, highly stereotyped limb movements, most frequently confined to the lower extremities. PLM can occur during sleep (PLM during sleep, PLMS) or in resting wake (PLM during wakefulness, PLMW). Typically there is an extension of the big toe, often combined with partial flexion of the ankle, the knee, and sometimes, the hip (American Acedamy of Sleep Medicine, 2014). A neurophysiologic and polygraphic study aimed to characterize the pattern of muscle involvement of PLMS showed that the muscles most frequently involved were the tibialis anterior (right 74 %; left 76 %), the gastrocnemius (right 66 %; left 54 %), the biceps femoris right 55 %; left 57 %), and the rectus femoris (right 36 %; left 49 %) (Provini et al., 2001). In most cases EMG activity started in the tibialis anterior muscles. Axial muscles and muscles of the upper limbs were involved in 14 % of cases (Provini et al., 2001). These data pose the basis for the selection of the muscles tibialis anterior for recording and scoring leg movements.

#### 4.3.2 Scoring criteria

Standards for recording and scoring leg movements are defined in the American Academy of Sleep Medicine (AASM) scoring manual (Berry et al., 2018) and by the World Association of Sleep Medicine (WASM) (Ferri et al., 2016). Leg movements (LM) are recorded in both legs through surface electrodes placed longitudinally and symmetrically in the middle of the anterior tibialis muscle so that they are 2–3 cm apart or 1/3 of the length of the anterior tibialis muscle (Berry et al., 2018). The AASM and WASM criteria for the identification of LMs and the definition of PLMs are reported in Table 3. The presence of PLMS is defined by a PLMS index > 15/h in adults and > 5/h in children.

**Table 3:** Simplified overview of the American Academy of Sleep Medicine (AASM) (Berry et al., 2018) and World Association of Sleep Medicine (WASM) (Ferri et al., 2016) criteria for the identification of leg movements (LMs) and the definition of periodic leg movements (PLMs).

	AASM (Berry et al., 2018)	WASM (Ferri et al., 2016)
LM		
Minimum duration	0.5 s	0.5 s
Maximum duration	10 s	None (but candidate LMs are those with a duration 0.5–10 s)
Timing of the onset	Point at which there is an 8 µV increase in EMG voltage above resting EMG	Point at which there is an 8 µV increase in EMG voltage above resting EMG
Timing of the ending	Start of a period lasting at least 0.5 s during which the EMG does not exceed 2 µV above resting EMG 8 µV above resting EMG for at least 0.5 s	Start of a period lasting at least 0.5 s during which the EMG does not exceed 2 µV above resting EMG
Minimum amplitude	NA	NA
Morphology threshold		The event contains a period ≥ 0.5 s with the median EMG amplitude ≥ 2 µV above resting baseline

PLM	At least 4 consecutive LMs	At least 4 candidate LM
Intermovement interval	5–90 s	10–90 s
End of PLM sequence	NA	Candidate LM intermovement interval < 10 s or > 90 s *OR* LM that are not CLM
Association with arousal	If they occur simultaneously, overlap, or when there is < 0.5 s between the end of one event and the onset of the other event	If they are overlapping or when there is < 0.5 s between the end of one event and the onset of the other event
Association with respiratory events	An LM should not be scored if it occurs 0.5 s preceding or following a respiratory event	Candidate LM that have some part overlapping with an interval of 2 s before to 10.25 s after the end of a respiratory event

CLM, candidate leg movement; EMG, electromyography.

#### 4.3.3 Alternative methods to assess PLMS

As reported above, the gold standard for detection of leg movements and quantification of PLMS is polysomnography with recording of the EMG activity in the tibialis anterior muscles. Alternative methods to measure PLMS have been developed and validated, including leg-worn actigraphy and 3D video detection based on artificial intelligence. Actigraphy has been extensively used, as it presents both economic and technical advantages compared to polysomnography and allows assessment of PLMS over several consecutive nights. However data show considerable heterogeneity, so that further research is needed to allow its use in both clinical and research settings (Plante, 2014). More recently developed methods to detect PLMS are based on artificial intelligence. A promising novel method is based on contactless monitoring of movements through a 3D time-of-flight camera (Seidel et al., 2020).

#### 4.3.4 Epidemiology

PLMS are observed in many sleep disorders. As reported above, they are found in more than 80 % of RLS patients (Montplaisir et al., 1997), and although not required for the diagnosis, their presence supports the diagnosis of RLS (American Acedamy of Sleep Medicine, 2014). PLMS are observed in other sleep disorders such as obstructive sleep apnoea syndrome (Manconi et al., 2014), narcolepsy (Dauvilliers et al., 2007), REM sleep behaviour disorder (Zhang et al., 2020) and other neurological disorders (e.g., movement disorders, multiple sclerosis, stroke, spinal cord injury) (Ferri et al., 2021, Högl and Stefani, 2017, Lee et al., 1996, Proserpio et al., 2015, Telles et al., 2012, Woo et al., 2017).

PLMS are also found among healthy sleepers (Frauscher et al., 2014, Pennestri et al., 2006).

#### 4.3.5 Periodic limb movement disorder

Periodic limb movement disorder (PLMD) is characterized by PLMS in conjunction with clinical sleep disturbance or fatigue that cannot be accounted for by another primary sleep disorder or another aetiology (American Acedamy of Sleep Medicine, 2014). The diagnostic criteria of the International Classification of Sleep Disorders are reported in Table 4.

**Table 4:** Criteria for the diagnosis of periodic limb movement disorder (PLMD) (American Academy of Sleep Medicine, 2014).

Criteria A-D must be met:
A) A polysomnography demonstrates PLMS, as defined in the most recent version of the American Academy of Sleep Medicine (AASM) Manual for the scoring of sleep and Associated Events.
B) The frequency is > 5/hour in children or > 15/hour in adults
C) The PLMS cause clinically significant sleep disturbance or impairment in mental, physical, social, occupational, educational, behavioural, or other important areas of functioning.^a^
D) The PLMS and the symptoms are not better explained by another current sleep disorder, medical or neurological disorder, or mental disorder (e.g., PLMS occurring with apneas or hypopneas should not be scored).^b^

PLMS, periodic leg movements during sleep.

a The presence of insomnia or hypersomnia with PLMS is not sufficient to establish the diagnosis of PLMD. Studies have shown that in most cases the cause of the accompanying insomnia or hypersomnia is something other than the PLNS. To establish the diagnosis of PLMD, it is essential to establish a reasonable cause-and-effect relationship between the insomnia or hypersomnia and the PLMS. This requires that other causes of insomnia such as anxiety or other causes of hypersomnia such as obstructive sleep apnea or narcolepsy are ruled out.

b PLMD cannot be diagnosed in the context of RLS, narcolepsy, untreated obstructive sleep apnea, or REM sleep behavior disorder; PLMS occur commonly in these conditions but the sleep complaint is more readily ascribed to the accompanying disorder. The diagnosis of RLS takes precedence over that of PLMD when potentially sleep-disrupting PLMS occur in the context of RLS. In such cases, the diagnosis of RLS is made and the PLMS are noted.

PLMD is thought to be rare, however the exact prevalence is unknown. Some predisposing factors have been described, such as a positive family history of RLS (American Acedamy of Sleep Medicine, 2014). This might be due to the presence of genetic risk factors associated with RLS and PLMS (Schormair et al., 2017, Tilch et al., 2020).

#### 4.3.6 Pathogenesis

Both spinal and supraspinal mechanisms have been reported to be involved in the generation of PLMS (Clemens et al., 2006). The role of spinal mechanisms is supported by data collected from patients with spinal cord injury, showing the presence of leg movements during sleep (Ferri et al., 2015). However, only a small subgroup of these patients shows leg movements (LMs) with a clear periodicity, so that it can be hypothesized that a genetic predisposition is required for the LMs to assume the periodic character (Ferri et al., 2015). For what concerns supraspinal neural substrates of PLMS, an EEG study showed an involvement of a large-scale motor network (which includes pericentral, dorsolateral prefrontal, and cingulate regions) (Kim et al., 2020). Moreover, part of the default mode network and motor control area (including the right inferior parietal, temporoparietal junction, and middle frontal regions) correlated with the PLMS index (Kim et al., 2020). Further research focused on better clarifying the pathogenetic mechanisms underlying PLMS is much needed, particularly in light of the potential clinical implications of PLMS (discussed in the next section).

#### 4.3.7 Clinical implications

The presence of PLMS in subjects without RLS might suggest a genetic risk of RLS. In fact, genetic risk factors have been associated with both RLS and PLMS (Schormair et al., 2017, Tilch et al., 2020).

A complex and dynamic interaction between PLMS, activity of the autonomic nervous system and arousals has been reported (Ferri et al., 2007). In RLS patients, an association between PLMS and an increase in both systolic and diastolic blood pressure has been reported (Pennestri et al., 2007, Siddiqui et al., 2007), and changes in blood pressure and heart rate in association with PLMS have been found also in healthy subjects (Pennestri et al., 2013). In line with these findings, activation of the sympathetic nervous system has been found in relation to PLMS (Guggisberg et al., 2007, Lin et al., 2020). Of note, PLM-related increases in heart rate (Winkelman, 1999) seem to be gender and age dependent, with a reduction in heart rate variability with age (Gosselin et al., 2003). Of note, in the large community-based sample of the *Osteoporotic Fractures in Men Study* an independent risk for myocardial infarction conferred by RLS and PLMS was found (Winkelman et al., 2017). Although supported by the above-mentioned studies, the potential role of PLMS as risk factor for cardiovascular events is still debated and more data are needed to definitely clarify this issue.

### 4.4 Discussion and summary

Restless legs syndrome (RLS) is a sensorimotor disorder described as an unpleasant sensation during rest with an urge to move. A spinal dopaminergic dysfunction has been suggested to be the underlying mechanism of the painful sensations occurring at night, which disappear with movement. Quantification of the abnormal movements can be standardized with help of the suggested immobilization test. Although subclinical sensory neuropathy has been proposed as RLS trigger, the coexistence of RLS and small fibre neuropathy seems to be rare. In up to 80 % of patients with RLS, periodic leg movements (PLM) during sleep are present which are repetitive, highly stereotyped limb movements. Polysomnography with EMG recording of the most commonly involved limb muscle, the tibialis anterior, is the gold standard for detection and quantification of periodic limb movements.

## 5 Support

None.

## Declaration of Competing Interest

The authors declare that they have no known competing financial interests or personal relationships that could have appeared to influence the work reported in this paper.
